# A glimpse into the future: modelling global prevalence of hypertension

**DOI:** 10.1186/s12889-023-16662-z

**Published:** 2023-10-03

**Authors:** Emmanuel B. Boateng, Ama G. Ampofo

**Affiliations:** 1 School of Health and Society, University of Wollongong, Wollongong, NSW 2522 Australia; 2 School of Medicine and Public Health, University of Newcastle, Callaghan, Australia

**Keywords:** Prevalence, Machine learning, Forecast, Hypertension, Sex

## Abstract

**Background:**

Hypertension is a major risk factor for cardiovascular diseases. Insights and foresights on trends of hypertension prevalence are crucial to informing health policymaking. We examined and projected the patterns of hypertension prevalence among sexes.

**Methods:**

Using annual hypertension prevalence (18 + years) data sourced from WHO Global Health Observatory data repository from 1975 to 2015, Prophet models were developed to forecast the 2040 prevalence of hypertension in males, females, and both sexes. We used k-means clustering and self-organising maps to determine the clusters of hypertension prevalence concerning both sexes among 176 countries.

**Results:**

Worldwide, Croatia is estimated to have the highest prevalence of hypertension in males by 2040, while that of females is in Niger. Among the world’s most populated countries, Pakistan and India are likely to increase by 7.7% and 4.0% respectively in both sexes. South-East Asia is projected to experience the largest hypertension prevalence in males, whereas Africa is estimated to have the highest prevalence of hypertension in females. Low-income countries are projected to have the highest prevalence of hypertension in both sexes. By 2040, the prevalence of hypertension worldwide is expected to be higher in the male population than in female. Globally, the prevalence of hypertension is projected to decrease from 22.1% in 2015 to 20.3% (20.2 – 20.4%) in 2040. We also identified three patterns of hypertension prevalence in 2040, cluster one countries are estimated to have the highest prevalence of hypertension in males (29.6%, 22.2 – 41.1%) and females (29.6%, 19.4 – 38.7%).

**Conclusion:**

These findings emphasise the need for new and effective approaches toward the prevention and control of hypertension in Africa, South-East Asia, and Low-income countries.

**Supplementary Information:**

The online version contains supplementary material available at 10.1186/s12889-023-16662-z.

## Introduction

Globally, hypertension is recognised as a major risk factor for cardiovascular diseases and stroke [1]. Hypertension also contributes to just over one-fifth of the population attributable fraction of cardiovascular diseases [1], and the leading risk factor for more than 10 million deaths and 218 million disability-adjusted life-years worldwide [2]. Between 2007–2017, the estimated years of lives lost from ischemic heart disease and stroke have increased by 17.3% and 12% respectively [3]. This makes hypertension a worldwide public health challenge [4]. Hypertension is known to be associated with non-modifiable risk factors such as sex [5–7]. However, the mechanisms of sex differences in hypertension prevalence remain unclear. Previous studies on the association between gender and blood pressure (BP) control have shown conflicting results, and hence may have contributed to this ambiguity. For example, some studies reported that women have better BP control than men, while others showed that there was no difference, and some found that men have effective BP control than women [8–12]. A plausible explanation for these outcomes could be attributed to the diverse age distributions among the study populations examined in such studies [5]. In 1947, men consistently recorded higher BP than women in the United States of America [13]. In 2015, the prevalence of hypertension was higher in men (24.1%) compared to women (20.1%) [14, 15]. Over the years, the increase in hypertension prevalence is still greater in men than in women [16]. However, after the onset of menopause, there is a flip with a rapid increase in the prevalence of hypertension in women compared to men [16–19].

While systematic analysis and multinational cohort studies have reported global trends of hypertension prevalence across various countries [1, 2, 20] and country-specific trends such as China [19, 21], there is limited studies on the projections of global hypertension prevalence. In 2005, a 60% increase in the prevalence of hypertension is expected among adults in 25 countries by 2025 [22]. This study aims to forecast the 2040 prevalence of hypertension considering sex differences across numerous countries. Hypertension prevalence estimates for the World Bank income groups, World Health Organisation (WHO) regions and worldwide were also provided. This study is novel as it uses machine learning techniques to identify the national and sectoral dynamics, trends, homogeneities, and heterogeneities in the prevalence of hypertension. Employing machine learning (ML) techniques are likely to contribute to a comprehensive synthesis of evidence to inform policy [23]. This study provides both insights and foresights for certain countries, regions, and income groups towards the development of national and international policies governing the prevention and control of hypertension.

## Methods

### Data description

We obtained hypertension (Raised BP, SBP >  = 140 OR DBP >  = 90, %, age-standardised estimate, 18 + years) data from WHO Global Health Observatory data repository (https://www.who.int/data/gho/data/indicators/indicator-details/GHO/raised-blood-pressure-(sbp-=140-or-dbp-=90)-(age-standardized-estimate). Annual data from 1975 to 2015 for hypertension prevalence of male (180 countries/territories; hereafter referred to as countries for simplicity), female (186 countries), both sexes (187 countries), the four World Bank income groups, and the six WHO regions were applied to the forecasting. While years’ 2010, 2015, and 2040 (projected) hypertension prevalence data for male and female were used for the clustering. Years’ 2010, 2015, and 2040 (projected) hypertension prevalence data for male and female were standardised to eliminate the possibilities of higher hypertension rates dominating the lower ones, hence creating a levelled-playing field [24, 25]. Standardisation is formulated as:

1 $$z=\frac{x-\mu }{\sigma }$$

where x is an observation of a variable (hypertension prevalence data on male or female) from the data in all years (2010, 2015, and 2040), µ is the mean of all observations of the variable from the data in all years, and σ is the standard deviation of all observations of the variable from the data in all years. Standardising the data is crucial to the models as it lessens the severe computations associated with neural networks [24].

### Statistical analyses

This study followed AG Ampofo and EB Boateng [26] approach in forecasting and clustering of time-series health data. Initially, we forecasted year 2040 hypertension prevalence for the countries, worldwide, the four World Bank income groups, and the six WHO regions. We used k-means clustering to provide insights on the number of clusters for years’ 2010, 2015, and 2040. Afterwards, we employed SOMs to independently fit each country’s hypertension prevalence onto grids of neurons by constructing low-dimensional maps. Before initialising the SOMs, we seeded the random number generator on each dataset to prevent the prospects of switching cluster labels [27, 28]. The cluster memberships per the SOMs groupings were cast onto geographical maps using choropleth mapping techniques. All clustering analyses were performed using RStudio v1.1.456, scientific python development environment (Spyder) v3.2.8 was used for executing all forecasting and geospatial map visualisations.

Prophet forecasting models were used to forecast year 2040 values for hypertension prevalence. This novel forecasting algorithm is employed by Facebook in producing high-quality forecasts. The model is robust to missing data, shifts in the trend, and mostly handles outliers efficiently [29]. Due to these benefits, Prophet has shown promising results in a variety of health-related scenarios. For example, Prophet has been used to forecast the numbers of deaths, recoveries and new infected cases due to Covid-19 following a number of weeks and months [30–33], the numbers of patients receiving outpatient or inpatient treatment for schizophrenia over the next 36 months [34], future suicide trend [35], and dissolved oxygen in diverse aquaculture environments [36]. For the Prophet model forecasting procedure in this study, the trend functions of all forecasts were set to logistic growth curves rather than linear trends to capture the natural variations of hypertension prevalence.

Considering the yearly data used in this study, we defined the seasonal argument of all models as “False”, and holidays as “None”. We maintained the default setting of the number of changepoints parameter, that is, Prophet determines 25 likely changepoints that are uniformly distributed in the first 80% of the data [29]. The default 1000 uncertainty samples were maintained, as fewer samples could lead to biases [26]. The default parameters are usually known to work well in delivering the best results [37]. To account for uncertainties in the forecasts, Prophet models presume that the mean frequency and magnitude of trend variations will be the same as that which was seen in the history [29]. Put simply, Prophet models assume that the future will have similar trend shifts as the past. The model then simulates the future rate changes that fit those of the past by replacing τ with a variance deduced from the data [37]. This parameter τ regulates the flexibility of the model in changing its rate [37]. When τ increases, the flexibility of the model increases to fit the past and so training error will decrease [26]. Suggesting that the simulated future patterns are used to compute the uncertainty intervals. The uncertainty intervals in this study were set to 95%, while preserving the 1000 uncertainty samples since smaller samples may lead to greater variances [26].

K-means clustering was used to find the dimensions of the rectangular node lattices, hence determining the underlying pattern of hypertension prevalence within years’ 2010, 2015, and 2040 datasets (for only male and only female). We experimented on 1–20 clusters and assessed their ratio of the sum of squares between clusters to the total sum of squares. Then, we followed the elbow method [38] by observing the ratios and kinks in their plots to evaluate saturation points as the optimal number of clusters. The proposed optimal number of clusters by the k-means algorithm was used to suggest the ideal number of neurons and hence applied to the development of the SOMs. We employed learning rates of 0.05 and 0.01, that is, to decline from a learning rate of 0.05 to 0.01 to prevent underfitting and overfitting. Upon prior experimentations, 200 iterations for each SOM were deemed effective in associating the data points as members of the specified number of clusters/neurons. We calculated the variable score, mean, minimum, and maximum hypertension rates within a cluster as its characteristics. The variable score is the normalised fan size which denotes the contribution of a variable in the cluster, where 0 = least contribution and 1 = highest contribution [26].

## Results

### Countries with the highest and least prevalence of hypertension in 2040

We forecasted the 2040 hypertension prevalence for male (180 countries), female (186 countries) and both sexes (187 countries), however for brevity, we show the first fifty countries with the highest prevalence of hypertension for each sex (Tables 1, 2 and 3). Each country is placed in their corresponding income group. The remaining forecasts are presented in the Supplementary material (pp 2–11).

**Table 1:** Projected top 50 highest hypertension prevalence countries/territories for 2040 (Male) and their income groups

Income group*	Overall rank	Country	2015	2040	∆%^b^	L^c^	U^c^	Income group	Overall rank	Country	2015	2040	∆%^b^	L^c^	U^c^
High-income								Low-income							
1	1	Croatia	38.4	41.1	7.1	36.8	45.4	1	3	Uganda	26.7	37.5	40.3	37.3	37.7
2	2	Trinidad and Tobago	27.6	40.8	47.8	40.5	41.1	2	9	Burundi	27.3	32.2	17.9	30.4	33.8
3	7	Romania	34.7	33.1	-4.7	28.9	37.1	3	11	Ethiopia	28.8	31.8	10.6	30.2	33.3
4	13	Slovenia	35.8	31.6	-11.7	27.9	35.4	4	14	Yemen	29.9	31.5	5.2	28.4	34.2
5	15	Lithuania	36.1	31.4	-13.1	26.3	37	5	16	Mali	31.2	31.2	0.1	28.9	33.3
6	34	Poland	34.6	29	-16.2	25.6	33.1	6	17	Somalia	33.5	31.1	-7.1	27.4	34.7
7	37	Slovakia	34.3	28.7	-16.3	26.4	31.2	7	18	Afghanistan	30.4	31.1	2.3	29	33.3
8	38	Antigua and Barbuda	26.4	28.6	8.3	25.8	31.4	8	19	Sudan	30.6	30.8	0.8	28.7	32.8
9	41	Czechia	34.4	28.2	-17.9	24.6	32.5	9	20	Malawi	27.8	30.7	10.4	29	32.4
10	42	Hungary	36.1	28.2	-21.8	26	30.8	10	22	Central African Republic	31.4	30.5	-2.9	28.7	32.3
11	43	Estonia	34.3	28.2	-17.8	24.9	32	11	23	Chad	31.6	30.4	-3.9	27.9	32.8
Lower-middle-income								12	25	Eritrea	28.2	30.2	7.2	29.1	31.5
1	6	Pakistan	31.5	34.6	9.7	33.3	35.7	13	27	Burkina Faso	31.3	29.8	-4.9	27.4	32
2	8	Nepal	29.7	33	11	31.6	34.4	14	32	Niger	31.3	29.1	-7	26.9	31.3
3	24	Vanuatu	24.2	30.3	25.2	30.1	30.4	15	47	Congo, the Dem. Rep	29.3	28	-4.4	26.1	30.1
4	26	Micronesia	26.6	29.8	12	27.9	31.6	16	48	Guinea	29	27.9	-3.8	26.3	29.4
5	28	Comoros	27.4	29.4	7.4	28.1	30.8	Upper-middle-income							
6	29	Bhutan	28.5	29.4	3.2	28.3	30.5	1	4	Bosnia and Herzegovina	34	37.1	9.1	33.7	40.3
7	30	Papua New Guinea	25.1	29.2	16.4	28.3	30.1	2	5	Moldova	33.6	34.8	3.6	31.1	38.5
8	33	Cambodia	26.3	29	10.4	27.1	30.8	3	10	Kazakhstan	30.4	31.8	4.8	28.7	34.7
9	36	Tajikistan	26.4	28.8	9.1	27.1	30.7	4	12	Saint Lucia	29.9	31.7	6	29.3	34.1
10	39	Tanzania	26.6	28.5	7.1	27.5	29.4	5	21	Albania	33	30.7	-7.1	28.4	33.2
11	44	Myanmar	24.9	28.1	12.9	27.9	28.3	6	31	Macedonia	32.7	29.2	-10.8	26.9	31.5
12	45	India	26.6	28.1	5.6	27.3	28.9	7	35	Montenegro	34.4	28.9	-16.1	24.5	33.4
13	49	Viet Nam	25	27.8	11.2	26.7	28.8	8	40	Bulgaria	33.6	28.4	-15.6	24.1	33.1
14	50	Kenya	26.5	27.6	4.2	25.6	29.6	9	46	Equatorial Guinea	29.2	28.1	-3.9	25.2	31.1

^a^Raised BP (SBP >  = 140 OR DBP >  = 90) (%, age-standardised estimate), 18 + years

^b^Percentage change in hypertension prevalence from 2015 to 2040

^c^Lower and upper confidence intervals (95%) for the 2040 forecast

^*^2022–2023 World Bank country classifications by income level

**Table 2:** Projected top 50 highest hypertension prevalence countries/territories for 2040 (Female) and their income groups

Income group*	Overall rank	Country	2015	2040	∆%^b^	L^c^	U^c^	Income group	Overall rank	Country	2015	2040	∆%^b^	L^c^	U^c^
Low-income								Lower-middle-income							
1	1	Niger	35.8	38.7	8.2	38.4	39	1	11	Nepal	29.5	32.7	11	31.6	33.8
2	2	Chad	33.8	38.6	14.1	35.9	40.8	2	13	Lesotho	30.8	32.5	5.4	31.1	33.9
3	3	Ethiopia	31.7	38.1	20.2	37.3	38.8	3	15	Eswatini	30.9	32.1	3.9	29.1	35.2
4	4	Burundi	31.1	37.3	19.9	36.3	38.3	4	16	Papua New Guinea	25.8	32	24.2	31.1	33
5	5	Mali	33.6	34.9	4	32.8	36.9	5	18	Comoros	28.2	31.1	10.4	28.9	33.2
6	6	Burkina Faso	33.2	34.7	4.6	32.7	36.8	6	20	Pakistan	29.5	30.9	4.9	30.4	31.5
7	7	Malawi	29.6	34.1	15.2	32.6	35.6	7	23	Timor-Leste	28.1	30.6	8.8	29.3	31.8
8	8	Afghanistan	30.7	33.5	9.1	32.8	34.2	8	25	Tanzania	27.7	29.2	5.5	27.9	30.5
9	9	Uganda	27.7	33	19.1	32.5	33.5	9	27	Kenya	26.7	28.9	8.1	27.5	30.2
10	10	Eritrea	29.5	32.8	11.1	31.9	33.6	10	30	Senegal	30.4	28.2	-7.3	26.1	30.5
11	12	Guinea	31.4	32.6	3.7	30.8	34.4	11	31	Bhutan	27.6	28.1	1.7	26.5	29.5
12	14	Somalia	32.2	32.2	0	30.2	34	12	32	Zimbabwe	29.2	28	-4.3	25.6	30.1
13	17	Mozambique	29.7	31.8	7.2	29.4	34	13	33	Cambodia	25.5	27.9	9.3	24.9	30.6
14	19	Sudan	29.6	31.1	5.1	29.2	33.1	14	35	Solomon Islands	23.6	27.5	16.5	26.7	28.2
15	21	Yemen	31.2	30.8	-1.3	28.9	32.7	15	36	Micronesia	23.2	27.5	18.4	25.9	29
16	22	Central African Republic	30.8	30.6	-0.6	29.2	31.9	16	37	Tajikistan	25.7	27.4	6.7	25.4	29.3
17	24	Rwanda	27.9	30.2	8.2	28	32.3	17	41	Vanuatu	24.1	26.8	11.3	25.7	28.2
18	26	Madagascar	28.2	29.1	3.2	28	30.2	18	42	Angola	29.6	26.8	-9.6	24.5	29.3
19	29	Guinea-Bissau	30.7	28.3	-7.7	25.4	31	19	45	Kyrgyzstan	25.7	25.8	0.5	24.5	27.2
20	34	Togo	29.2	27.7	-5.2	25.7	29.6	20	46	India	24.7	25.5	3.2	24.7	26.2
21	39	Sierra Leone	31	27.2	-12.2	24.3	30.3	21	50	Mauritania	31.4	25	-20.5	20.8	28.8
22	44	Congo, the Dem. Rep	27.6	26.5	-4.1	24.9	28.2	Upper-middle-income							
23	48	Liberia	28.3	25.4	-10.3	21.3	30.1	1	38	Saint Lucia	24.4	27.3	12	25.6	29
24	49	Zambia	26.5	25	-5.6	22.6	27.4	2	40	Equatorial Guinea	27.7	27	-2.4	25.2	28.9
High-income								3	43	Tonga	21.8	26.6	22.1	24.7	28.5
1	28	Trinidad and Tobago	23.9	28.4	18.8	26.8	29.7	4	47	Bosnia and Herzegovina	27.6	25.4	-8	22.4	28.6

^a^Raised BP (SBP >  = 140 OR DBP >  = 90) (%, age-standardised estimate), 18 + years

^b^Percentage change in hypertension prevalence from 2015 to 2040

^c^Lower and upper confidence intervals (95%) for the 2040 forecast

^*^2022–2023 World Bank country classifications by income level

**Table 3:** Projected top 50 highest hypertension prevalence countries/territories for 2040 (Both sexes) and their income groups

Income group*	Overall rank	Country	2015	2040	∆%^b^	L^c^	U^c^	Income group	Overall rank	Country	2015	2040	∆%^b^	L^c^	U^c^
Low-income								Lower-middle-income							
1	1	Chad	32.9	34.9	6.2	32.6	37.2	1	6	Pakistan	30.5	32.9	7.7	31.8	33.9
2	2	Burundi	29.2	34.9	19.6	33.6	36.1	2	10	Nepal	29.4	32.2	9.4	30.8	33.4
3	3	Niger	33.4	34	1.8	32.4	35.5	3	18	Papua New Guinea	25.6	30.6	19.6	29.6	31.5
4	4	Mali	32.6	33.2	1.7	31.4	34.8	4	20	Comoros	27.9	30.5	9.2	29	31.9
5	5	Malawi	28.9	32.9	13.7	31.6	34.1	5	24	Eswatini	29.8	29.4	-1.3	25.6	33.1
6	7	Uganda	27.3	32.8	20.1	31.9	33.6	6	25	Lesotho	29	29.4	1.3	28.1	30.8
7	8	Burkina Faso	32.6	32.7	0.3	30.2	35.1	7	26	Tanzania	27.3	29	6.2	28.1	29.9
8	9	Afghanistan	30.6	32.4	5.8	31.1	33.7	8	27	Timor-Leste	27.6	28.8	4.4	27.2	30.5
9	11	Somalia	32.9	31.9	-3.1	29.5	34.3	9	28	Micronesia	25	28.8	15.3	27.3	30.3
10	12	Eritrea	29.1	31.6	8.7	33	50	10	30	Cambodia	26.1	28.7	9.8	26.1	31
11	13	Yemen	30.7	31.6	2.9	28.3	34.9	11	31	Bhutan	28.1	28.6	1.9	27.2	30
12	16	Sudan	30.2	31.1	3	29.1	33	12	32	Tajikistan	26.1	28.5	9.1	26.4	30.6
13	17	Central African Republic	31.2	30.7	-1.6	29.1	32.3	13	33	Kenya	26.7	28.4	6.4	27	29.8
14	21	Guinea	30.3	30.3	0.1	28.7	32	14	41	Angola	29.7	27	-9.1	24.9	29.2
15	22	Mozambique	29.1	30	3	28	31.9	15	43	India	25.8	26.8	4	26	27.6
16	34	Rwanda	26.7	28.2	5.6	25.2	30.9	16	44	Senegal	30.2	26.7	-11.5	24.4	29.1
17	35	Madagascar	28.1	28.1	0.1	26.9	29.5	17	45	Vanuatu	24.2	26.7	10.3	25.1	28.4
18	37	Guinea-Bissau	30.3	27.7	-8.7	25.6	29.7	18	46	Kyrgyzstan	26.7	26.7	0	25.4	28
19	40	Congo, the Dem. Rep	28.5	27.4	-3.8	25.8	29.2	19	50	Zimbabwe	28.2	26	-7.6	24.3	28
20	48	Togo	28.9	26.2	-9.4	24	28.3	Upper-middle-income							
21	49	Sierra Leone	30.3	26.1	-13.9	23.1	29.1	1	15	Bosnia and Herzegovina	30.8	31.3	1.8	26.5	35.8
High-income								2	23	Saint Lucia	27.1	29.6	9.1	27.3	31.6
1	14	Croatia	32.4	31.5	-2.7	28.1	35.5	3	29	Moldova	29.8	28.7	-3.8	25.6	32
2	19	Trinidad and Tobago	25.8	30.5	18.1	29.1	31.8	4	36	Tonga	23.7	28.1	18.5	26.1	30
3	38	Romania	30	27.5	-8.3	24.7	30.1	5	39	Equatorial Guinea	28.4	27.4	-3.4	24.7	30.3
4	47	Slovenia	30.5	26.3	-13.8	23.1	29.8	6	42	Georgia	26.3	26.9	2.1	24.6	29.4

^a^Raised BP (SBP >  = 140 OR DBP >  = 90) (%, age-standardised estimate), 18 + years

^b^Percentage change in hypertension prevalence from 2015 to 2040

^c^Lower and upper confidence intervals (95%) for the 2040 forecast

^*^2022–2023 World Bank country classifications by income level

For males, Croatia is projected to have the highest hypertension prevalence, increasing from a prevalence of 38.4% in 2015 to 41.1% in 2040. On the other hand, the least prevalence of hypertension in males is projected to be in the United Kingdom (5.5%). The largest rise of 47.8% is expected to be in Trinidad and Tobago, and the biggest decline (69.0%) is projected to be in the United Kingdom. On the African continent, Uganda (37.5%) is likely to record the highest prevalence of hypertension in males. In Asia, Pakistan (34.6%) will have the peak prevalence of hypertension. The biggest prevalence of hypertension in Europe is expected to occur in Croatia (41.1%). Trinidad and Tobago (40.8%) are projected to have the highest hypertension prevalence in North America. The second highest prevalence in North America will be recorded in Saint Lucia (31.7%). Argentina (22.7%) is likely to experience the highest prevalence of hypertension in South America. Vanuatu (30.3%) is projected to have the highest male hypertension prevalence in Oceania. By 2040, no country in South America was found in the 90 highest hypertension prevalence countries.

For females, Niger is likely to have the peak prevalence of hypertension by 2040, rising from a prevalence of 35.8% to 38.7%. In contrast, Singapore (3.9%) will have the least prevalence of hypertension, representing 65.7% decline since 2015. The biggest increase (24.2%) in hypertension prevalence in females will be in Papua New Guinea. Somalia is estimated to maintain its 2015 prevalence in 2040. The largest decline (69.3%) in hypertension prevalence is likely to occur in the Netherlands. On each continent, the highest prevalence of hypertension in females is estimated to occur in Niger (Africa), Afghanistan (Asia), Papua New Guinea (Oceania), Trinidad and Tobago (North America), Bosnia and Herzegovina (Europe), and Guyana (South America). No country in South America was among the fifty highest hypertension prevalence countries.

Overall, for both sexes, Chad (34.9%) is projected to have the highest prevalence of hypertension, while the least prevalence is likely to occur in the United Kingdom (5.7%). Uganda is estimated to have the biggest increase (20.1%) in hypertension prevalence, and the largest decrease (64.7%) is expected to be in the Netherlands. On each continent, the peak prevalence of hypertension in both sexes is likely to be recorded in Chad (Africa), Pakistan (Asia), Croatia (Europe), Papua New Guinea (Oceania), Trinidad and Tobago (North America), and Guyana (South America). No country in South America was among the sixty highest hypertension prevalence countries. The fitted curves of these forecasts are shown in the Supplementary material (pp 12–35).

### Projections of hypertension prevalence in 2040 for the most populated countries

The hypertension prevalence of both sexes for the ten most populated countries are presented in Table 4. By 2040, Pakistan is likely to have the highest growth of 7.7% since 2015 among the ten largest populated countries. This increase in Pakistan’s hypertension prevalence suggests a rise from 30.5% in 2015 to 32.9% in 2040. The hypertension prevalence in Nigeria is projected to decrease from 23.9% in 2015 to 15.3% in 2040. India is projected to have the second highest hypertension prevalence among the ten most populated countries. Hypertension prevalence in the USA is expected to decrease from 12.9% in 2015 to 11.5% in 2040. The hypertension prevalence in China is projected to decline from 19.2% in 2015 to 15.3% in 2040. The remaining forecasts are available in Table 4, and fitted curves are presented in the Supplementary material (pp. 28 – 35).

**Table 4:** 2040 projected hypertension prevalence for top 10 populated countries (Both sexes)

No.^+^	Country*	2015^a^	2040^a^	∆%^b^	L^c^	U^c^
1	China	19.2	15.3	-20.4	12.4	18.2
2	India	25.8	26.8	4.0	26.0	27.6
3	United States	12.9	11.5	-10.7	11.5	11.6
4	Indonesia	23.8	20.4	-14.3	18.5	22.3
5	Pakistan	30.5	32.9	7.7	31.8	33.9
6	Brazil	23.3	15.5	-33.3	14.0	17.2
7	Nigeria	23.9	15.3	-35.9	11.4	19.7
8	Bangladesh	24.7	22.4	-9.4	20.0	25.1
9	Russian Federation	27.2	20.0	-26.5	18.2	22.5
10	Mexico	19.7	14.3	-27.5	13.2	15.5

^a^Raised BP (SBP >  = 140 OR DBP >  = 90) (%, age-standardised estimate), 18 + years

^b^Percentage change in hypertension prevalence from 2015 to 2040

^c^Lower and upper confidence intervals (95%) for the 2040 forecast

^*^Total population ranking for 2021 (The World Bank Group, 2022)

^+^Number ranking is in terms of population size

### Projections of regional, income, and global prevalence of hypertension in 2040

By 2040, South-East Asia is estimated to have the highest prevalence of hypertension in males, with a 0.8% increase since 2015. Africa is likely to record the highest hypertension prevalence in females, representing a decline of 7.1% since 2015. Overall, for both sexes, South-East Asia will have the peak hypertension prevalence rising from 25.1% in 2015 to 25.3% in 2040. South-East Asia is also projected to record the biggest increase in hypertension prevalence in both sexes.

Concerning Income groups, Low-income countries are projected to have the highest prevalence of hypertension in males (28.4%), females (30.1%), both sexes (29.4%). In addition, the largest rise in the prevalence of hypertension is likely to occur in Low-income countries. On the other hand, High-income countries are estimated to have the least prevalence of hypertension in males (16.5%), females (9.7%), and both sexes (13.6%).

**Fig. 1:**
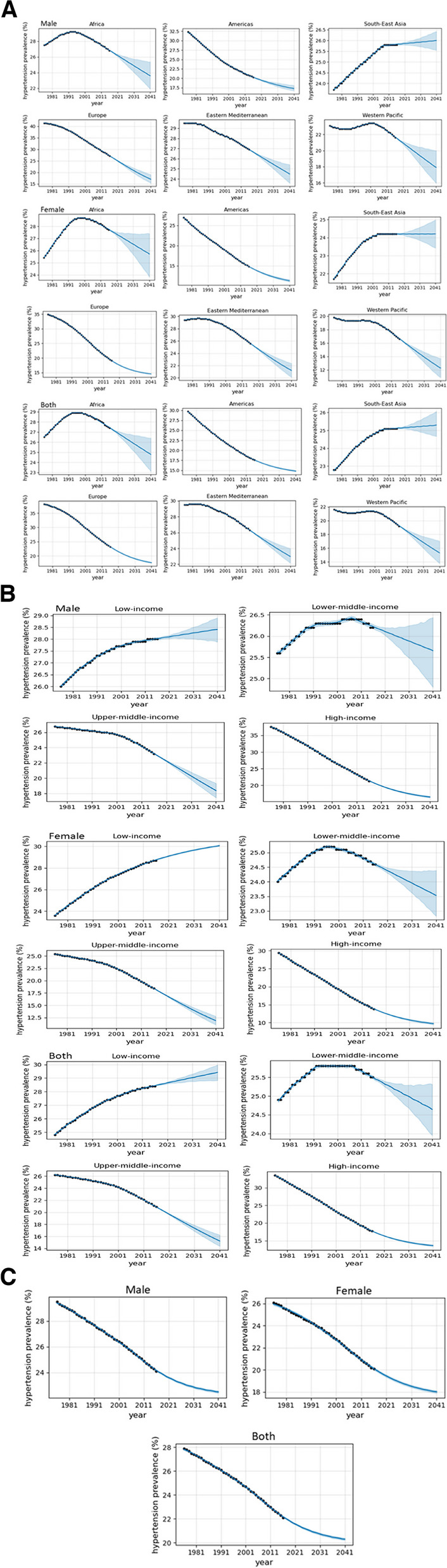
**a **Plot of actual data (dotted) and fitted curve (at 95% CI) for 2040 hypertension prevalence for regional groups. **b **Plot of actual data (dotted) and fitted curve (at 95% CI) for 2040 hypertension prevalence for income groups. **c** Plot of actual data (dotted) and fitted curve (at 95% CI) for 2040 global prevalence of hypertension

Globally, the prevalence of hypertension in 2040 is expected to be higher in males than in females. Also, the biggest proportional decline in the prevalence of hypertension will occur in the female population. Overall, the prevalence of hypertension in both sexes will decrease from 22.1% in 2015 to 20.3% in 2040, representing an 8.2% decrease. The remaining forecasts are available in Table 5 and fitted curves for all forecasts are presented in Fig. 1a, b, and c.

**Table 5:** Projected prevalence of hypertension at the regional, income, and global levels for 2040

Sex	World Bank Income Group	2015^a^	2040^a^	∆%^b^	L^c^	U^c^	Sex	WHO Region	2015^a^	2040^a^	∆%^b^	L^c^	U^c^
Male							Male						
	Low-income	28.0	28.4	1.5	27.9	28.9		Africa	26.8	23.6	-11.9	21.9	25.4
	Lower-middle-income	26.2	25.7	-2.1	24.8	26.4		Americas	20.3	17.3	-15.0	16.7	18.1
	Upper-middle-income	23.2	18.4	-20.8	17.4	19.3		South-East Asia	25.8	26.0	0.8	25.5	26.4
	High-income	21.3	16.5	-22.7	16.2	16.8		Europe	27.2	17.0	-37.5	15.3	19.0
Female								Eastern Mediterranean	26.9	24.5	-9.0	23.6	25.4
	Low-income	28.7	30.1	4.7	30.0	30.1		Western Pacific	21.6	17.9	-17.1	15.9	20.0
	Lower-middle-income	24.6	23.5	-4.3	22.8	24.4	Female						
	Upper-middle-income	18.4	11.9	-35.2	11.1	12.8		Africa	27.7	25.7	-7.1	23.8	27.4
	High-income	13.8	9.7	-29.4	9.5	10.0		Americas	14.8	11.3	-23.8	11.0	11.6
Both								South-East Asia	24.2	24.2	0.0	23.4	25.0
	Low-income	28.4	29.4	3.6	28.8	30.0		Europe	19.1	14.6	-23.7	14.5	14.6
	Lower-middle-income	25.5	24.6	-3.4	24.0	25.3		Eastern Mediterranean	25.6	21.2	-17.2	20.1	22.4
	Upper-middle-income	20.9	15.2	-27.1	14.4	16.2		Western Pacific	16.6	12.3	-26.0	10.9	13.7
	High-income	17.7	13.6	-23.1	13.4	13.9	Both						
Global								Africa	27.4	24.8	-9.6	23.1	26.4
	Male	24.1	22.5	-6.7	22.4	22.6		Americas	17.6	14.8	-15.9	14.7	14.9
	Female	20.1	18.0	-10.4	17.9	18.1		South-East Asia	25.1	25.3	0.8	24.7	26.1
	Both	22.1	20.3	-8.2	20.2	20.4		Europe	23.2	17.6	-24.2	17.5	17.7
								Eastern Mediterranean	26.3	23.0	-12.6	22.2	24.0
								Western Pacific	19.2	15.3	-20.3	13.8	17.0

^a^Raised BP (SBP >  = 140 OR DBP >  = 90) (%, age-standardised estimate), 18 + years

^b^Percentage change in hypertension prevalence from 2015 to 2040

^c^Lower and upper confidence intervals (95%) for the 2040 forecast

### Hypertension prevalence clusters

**Fig. 2:**
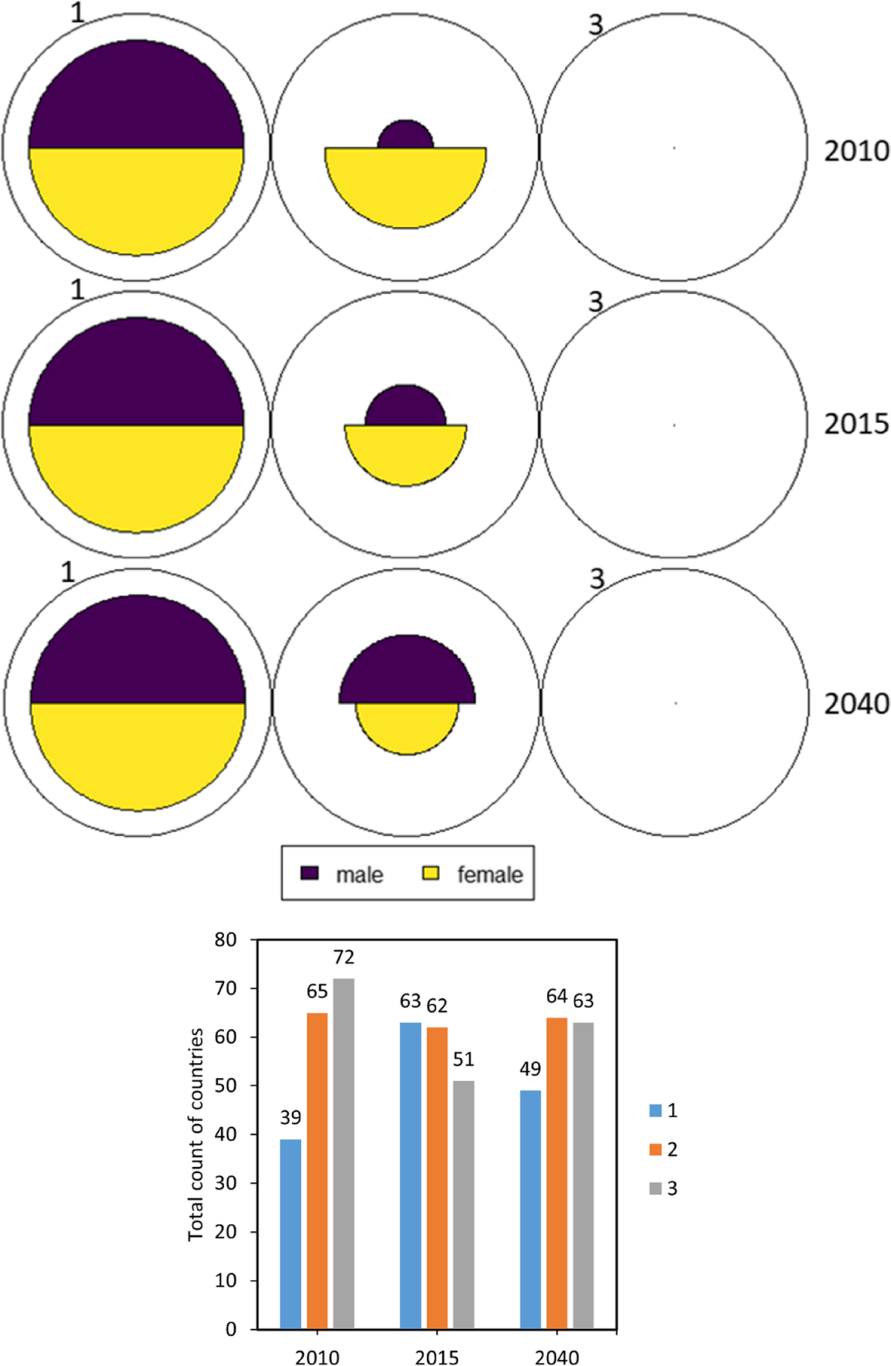
SOMs of hypertension prevalence and number of countries in each cluster

**Fig. 3:**
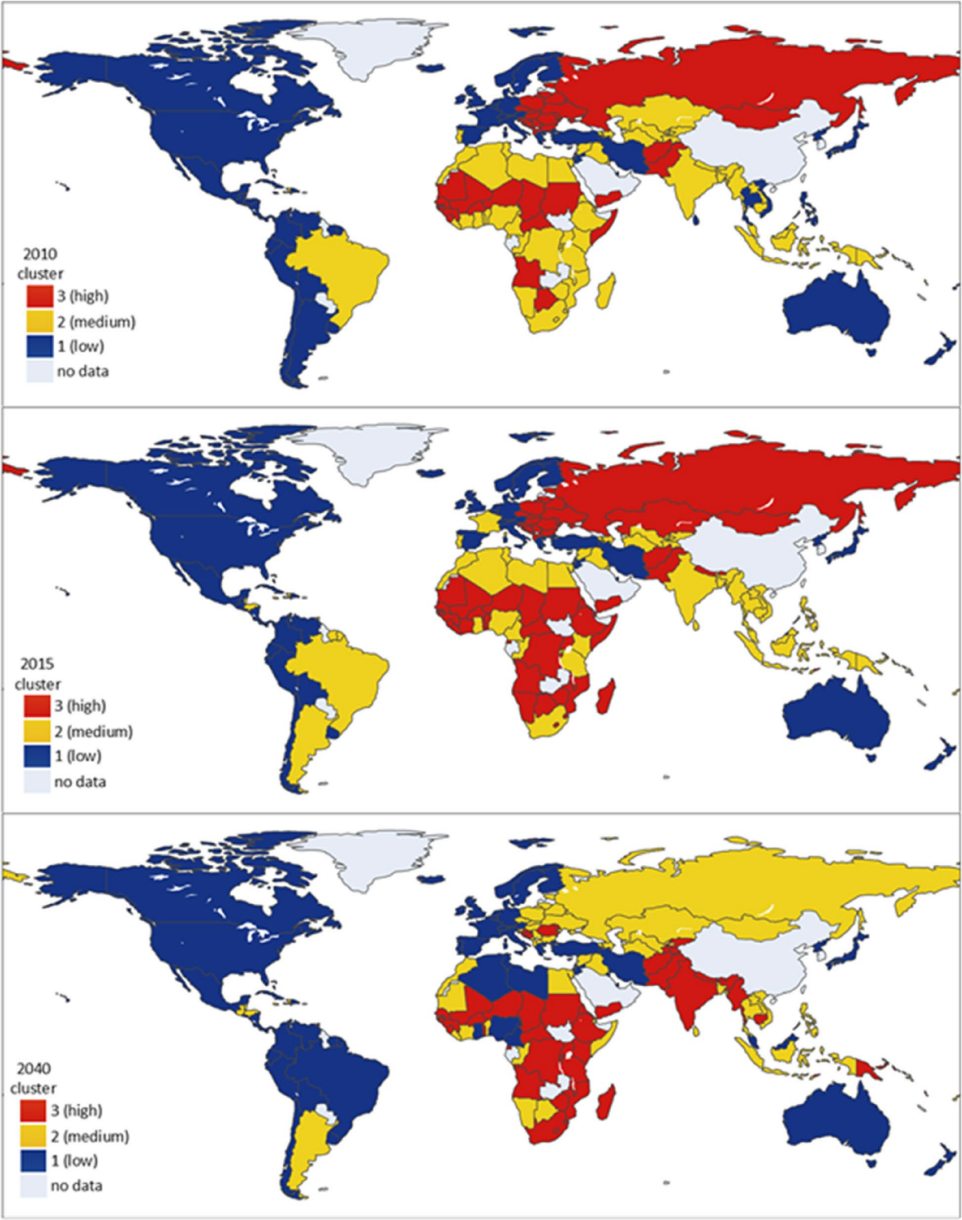
Geospatial distribution of the three hypertension prevalence clusters based on adult male and female hypertension prevalence for 176 countries/territories

Three clusters were deemed optimal to explain the variability in years’ 2010, 2015, and 2040 datasets. Additional clusters did not improve the explained variances of the models. For example, the initial three clusters in years’ 2010, 2015, and 2030 accounted for an average percentage increase of 23.7%, 24.4%, 20.7% in the data variance respectively, whereas the remaining 17 clusters explained an average percentage increase of 2.2%, 1.8%, and 1.5% respectively. Therefore, three neurons were used to configure the dimensions of the SOMs. We specified and trained three 3 × 1 two-dimensional lattices over 200 iterations. Fig. 2 presents the SOMs for the 176 countries according to their prevalence of hypertension similarities and disparities. Each circle signifies a cluster/pattern, with the fan sizes showing the proportion of hypertension prevalence in each sex. The bar graph in Fig. 2 provides details on the number of countries in each cluster. Countries in each cluster have also been specified in the Supplementary material (pp 38–41) and geographically shown in Fig. 3.

The highest hypertension prevalence cluster in years’ 2010, 2015, and 2040 is cluster one, with a variable score of 1.0 in both males and females. By 2040, countries in this cluster are projected to have an average hypertension prevalence of 29.6% (22.2 – 41.1%) in males and 29.6% (19.4 – 38.7%) in females. Forty-nine countries are expected to be in cluster one including Afghanistan, Angola, Bhutan, Burkina Faso and Eswatini. Eighteen countries (such as Yemen, Sudan, Sierra Leone, and Senegal) maintained their cluster memberships throughout the three years. By 2040, no country in the least hypertension prevalence cluster, i.e., cluster three, transitioned to cluster one.

Cluster two is the next highest hypertension prevalence cluster, with variable scores of 0.6 for male, and 0.5 for female. This cluster is an evolved cluster characterised by a moderate prevalence of hypertension, with the major contributor to hypertension prevalence gradually shifting from females to males. For instance, though, in 2010, 2015, and 2040, the prevalence of hypertension seemed to be consistently higher in males than females, the variable score, which shows the relative contribution of each sex’s hypertension prevalence among other sex’s in a particular cluster across the three years, indicates that the variable score of female hypertension prevalence decreased from 0.8 in 2010 to 0.6 in 2015, and then 0.5 in 2040. On the other hand, the variable score of male hypertension prevalence increased from 0.3 in 2010 to 0.4 in 2015, and then 0.6 in 2040 (Fig. 2 and Table 6). This shift in cluster behaviour is attributed to the dynamic change of time leading to the formation of novel/updated clusters to accommodate the prevailing or evolved cluster properties [26]. These dynamics associated with time-series clustering creates moving, emerging, or dying clusters [39, 40]. Nevertheless, some cluster properties are recognised over time [41]. For instance, a moderate prevalence of hypertension is a clear differentiating factor in cluster two. By 2040, this cluster is estimated to have an average prevalence of 24.8% (19.6 – 31.8%) in males and 19.5% (11.3 – 32.2%) in females. Seventeen countries including Armenia, Azerbaijan, Bangladesh, Barbados, and Congo have consistently been members of this cluster in the three years.

**Table 6:** Characteristics and variable scores of hypertension prevalence clusters

Year 2010		
Cluster	Male^a^	Female^a^
1	33.2 (vs = 1.0)	28.3 (vs = 1.0)
	[29.2–38.1]	[22.5–35.2]
2	27.2 (vs = 0.3)	26.0 (vs = 0.8)
	[23.8–31.5]	[21.8–30.5]
3	24.5 (vs = 0.0)	18.5 (vs = 0.0)
	[15.9–31.0]	[11.2–22.8]
Year 2015		
Cluster	Male^a^	Female^a^
1	30.8 (vs = 1.0)	27.8 (vs = 1.0)
	[26.1–38.4]	[20.9–35.8]
2	25.6 (vs = 0.4)	22.8 (vs = 0.6)
	[20.4–29.3]	[16.4–27.9]
3	22.1 (vs = 0.0)	15.7 (vs = 0.0)
	[15.3–27.8]	[10.5–20.4]
Year 2040		
Cluster	Male^a^	Female^a^
1	29.6 (vs = 1.0)	29.6 (vs = 1.0)
	[22.2–41.1]	[19.4–38.7]
2	24.8 (vs = 0.6)	19.5 (vs = 0.5)
	[19.6–31.8]	[11.3–32.2]
3	15.0 (vs = 0.0)	9.9 (vs = 0.0)
	[5.5–19.8]	[3.9–18.6]

^a^Raised BP (SBP >  = 140 OR DBP >  = 90) (%, age-standardised estimate), 18 + years

Note, vs is the variable score

The least hypertension prevalence cluster is cluster three, with the lowest variable score of 0.0 for both male and female. As such, the contribution of these sexes in this cluster is low, and the prevalence of hypertension continues to decrease in the cluster. For instance, in 2010, cluster three had the lowest prevalence of hypertension in males (24.5%, 15.9 – 31.0%) and females (18.5%, 11.2 – 22.8%). Similarly, in 2015, the hypertension prevalence in males (22.1%, 15.3 – 27.8%) and females (15.7%, 10.5 – 20.4%) was the lowest. By 2040, we project that the average prevalence of hypertension in males and females in cluster three would be 15.0% (5.5 – 19.8%) and 9.9% (3.9 – 18.6%) respectively. Most countries (49 out of 63) including Andorra, Australia, Belgium, Canada, Denmark, New Zealand, the United Kingdom, and the United States have consistently maintained their memberships with cluster three in 2010, 2015, and 2040. Further details of these clusters are presented in Table 6 and Supplementary material (pp 38–41).

## Discussion

This is the first study to project the prevalence of hypertension from 2015 to 2040 for over 180 countries, the four income groups, the six WHO regions, and the world concerning sex. Additionally, the present study examined and clustered the countries according to their prevalence of hypertension and sex. Three patterns of hypertension prevalence were identified, with cluster one having the highest prevalence of hypertension in adult males and females by 2040. Findings from this study may serve as a template for global action for control of hypertension in the future.

Our projections for 2040 suggest that the global prevalence of hypertension will decrease for both sexes. We also observed a projected decline in hypertension prevalence among highly populated countries in the world including China. However, India and Pakistan are likely to experience an increase among both sexes. In contrast, Kearney et al. [22] reported that the world will experience an increase in hypertension prevalence in the adult population by 2025, China was also projected to increase. A possible explanation for this finding could be attributed to the dynamic change of time (different forecast years), nuances in data sources and world classifications, and the number of observations. For instance, they used data from 1980 to 2002 where the 1993 world bank development reports, definitions and prevailing economic situations were different from present times [22]. Additionally, their study projected the prevalence of hypertension into 2025 for aged 20 years and older whereas our study forecasted into 2040 for adults aged 18 years and older [22]. Moreover, the input data and methods of estimating were dissimilar in both studies [22]. In line with existing studies [22], India will experience an increase in hypertension prevalence. In Pakistan, current estimates from 2016–2017 population-based study has already seen an increase in hypertension prevalence, with more than 40% of the participants reporting hypertension [42].

We also estimated that, globally, females are likely to experience the largest decline in hypertension prevalence compared to males. A particularly biggest decline (by more than 20%) will be experienced in High-income countries, Upper-middle-income countries, Europe, and the Western Pacific by 2040. For instance, the female population in the Netherlands is expected to experience the largest decline by 2040 which corroborates with a report by the World Health Organisation [14], where a substantial reduction in hypertension prevalence began in the year 2000 with a further decline by 2025. For the male population, Croatia is estimated to have the highest prevalence of hypertension by 2040. This finding is in line with recent studies on the increasing prevalence of hypertension in Croatia [43, 44]. We found that while Trinidad and Tobago is likely to record a high increase, the United Kingdom will experience a high reduction by 2040. The leading cause of death in Trinidad and Tobago is heart disease [45], accounting for 32% of all deaths in 2014 [46]. In contrast to our findings, studies have reported that hypertension prevalence has decreased in the United Kingdom since 2004 [47] and has been projected to further decline by 2025 [48].

Large economic and geographical disparities in hypertension prevalence were estimated and our findings indicate Low-income and African countries will be greatly impacted by hypertension by 2040. While Upper-middle and High-income countries is predicted to experience the largest decline in hypertension prevalence, Low-income countries will experience the highest increase in hypertension prevalence by 2040 in males, females and both sexes. Countries in Africa are likely to record the highest prevalence of hypertension, with Chad having the peak prevalence and Uganda recording the biggest increase by 2040 among both men and women. Females in Niger will also record the highest prevalence of hypertension. Previous studies have observed an increasing growth in the prevalence of hypertension in Low-income countries [15, 49] with a projected increase of over 25% by 2030 in Africa [50]. Other reports also indicate a high hypertension prevalence in Africa among adults above 55 years [51] and the prevalence nearly doubled between 1990–2010 [50]. We also report that South-East Asia is estimated to have a rise in hypertension rates, which corroborates with previous projected estimates [22].

Our findings suggest that since 2010 while High-income countries such as Australia and Denmark have consistently reported low prevalence of hypertension in cluster three, some Lower-middle-income countries in cluster one such as Angola and Eswatini have reported high prevalence for both men and women. Previous studies have recorded similar trends where there was decreasing hypertension prevalence in High-income countries and increasing prevalence among Low and Middle-income countries [15, 52, 53]. The decrease in hypertension prevalence occurring in Western countries is not surprising. Obviously, Western populations have had more time to adapt to a Western lifestyle and in most studies, also have the most negligible impact on cardiovascular health. In contrast, Indigenous people suffer the most cardiovascular health consequences from changing to a Western lifestyle.

The projected significant increase in the prevalence of hypertension in Africa, South-East Asia, and Low-income countries could be explained by the high consumption of salt [54], low consumption of fruits and vegetables [55–57], lack of low treatment of hypertension [20, 58] and insufficient physical activities [59], high economic growth and ageing population [15]. Therefore, effective primary interventions in line with the United Nations and WHO recommendation should be considered including reducing dietary sodium/salt intake alcohol consumption, smoking and tobacco use and increasing physical activities [60]. Additionally, recent guidelines for the management of hypertension including risk evaluation, treatment strategies (lifestyle advice and medications to address hypertension) should be implemented [61]. Continuous implementation of these strategies is likely to contribute to global efforts to reduce the prevalence of hypertension by 2040. Effective implementation of these interventions in Australia, Europe, and North America could account for the significant decline in cardiovascular mortality. There is a need to increase research investments into the high rates of hypertension in Africa, South-East Asia, and Low-income countries to prevent future complications of the disease. Budgetary allocations should be prioritised in these countries for health planning toward improvement in health infrastructure and human resources to manage and control hypertension.

### Strengths and limitations

With 40 years’ worth of time-series data, our study captures more historical and current trends suitable for high-quality long-term forecasts. However, our study is not without limitations. Similar to forecasting studies, the assumptions for cap and floor arguments in developing each Prophet model may slightly limit our results. Nevertheless, numerous simulations indicate this effect is minimal. Furthermore, due to the paucity of data for some countries, we could not provide estimates for those countries, as such, limits our ranking of highest hypertension prevalent countries. While we examined an enormous potential number of clusters, we had to determine this range of clusters earlier on, then evaluate each to identify the optimal cluster. This suggests a certain degree of subjectiveness in the choice of a representative cluster for each year. However, k-means clustering ensures convergence and efficiency in segmenting a dataset to determine a suitable cluster.

In a global manner, we applied a common method of standardisation across all features in all years’ datasets. Other types of standardisation may tend to be effective in clustering a specific year’s dataset. Also, our projections of the number of people with hypertension by 2040 is probably an underestimate since it does not account for the rapid changes in lifestyle [62, 63], and the introduction of cheap therapeutic interventions that could spread across the globe, and social determinants of hypertension. Considering that shifts in population have elevated cardiovascular risk factors [64], our findings do not take into account the gradual effect of migration on hypertension. As a result, the outcomes of this study should be interpreted in light of this. Likewise, considering that hypertension is multifactorial [65], and that the aetiologies that drive the prevalence of hypertension vary over time including nutrition, environmental stressors and behavior [65, 66], our estimates may be limited in capturing these causal changes of hypertension prevalence. There were variations in data collection and collation methods used in producing the WHO source data, which may have resulted in an overestimation or underestimation of our projections. For example, different blood pressure measurement tools were used including a mercury sphygmomanometer and digital oscillometric devices [15]. However, the Prophet model is known to outperform any other forecasting approach in diverse situations including its efficiency in handling missing data, shifts in trends and outliers [29].

Methodologically, this research contributes to the promising field of ML techniques in epidemiological studies. The lack of representative data for national and regional levels on the relationship between socioeconomic status variables and hypertension in Low and Middle-income countries has resulted in contradictory results [67]. While we did not directly use socioeconomic indicators, our results on regional and income group projections coupled with the country-specific analyses provide relevant evidence on the association between economic factors and hypertension and contributes to closing this research gap.

## Conclusion

Economic and geographical disparities in hypertension prevalence are projected to occur with the projected increase in the hypertension in Low-income countries, Africa, and South-East Asia. Government and multinational organisations should enhance population-based primary prevention strategies including education about hypertension risk factors. In addition, primary healthcare systems should be strengthened to offer individual lifestyle management and treatment in combination with population-based approaches to provide tailored care for people and the community at large. In Low-income countries where health-care resources are scarce, investment in these strategies could yield the greatest benefit. There is the need to increase research efforts to identify appropriate low cost and effective prevention interventions of hypertension to reduce the impact of hypertension by 2040.

### Supplementary Information

**Additional file 1: Table A1.** Projected hypertension prevalence for 2040 (Male). **Table A2.** Projected hypertension prevalence for 2040 (Female). **Table A3.** Projected hypertension prevalence for 2040 (Both). **Table A4.** Changes in hypertension prevalence cluster patterns. **Fig. A1a**. A1h. Plot of actual data (dotted) and fitted curve (95% CI) for hypertension prevalence (Male). **Fig. A2a.** A2h. Plot of actual data (dotted) and fitted curve (95% CI) for hypertension prevalence (Female). **Fig. A3a.** A3h. Plot of actual data (dotted) and fitted curve (95% CI) for hypertension prevalence (Both). **Fig. A4a.** Plot of actual data and fitted curve (95% CI) for hypertension prevalence (Income groups). **Fig. A4b.** Plot of actual data and fitted curve (95% CI) for hypertension prevalence (Regions).

## Data Availability

The datasets analysed during the current study are available in the WHO Global Health Observatory data repository, https://www.who.int/data/gho/data/indicators/indicator-details/GHO/raised-blood-pressure-(sbp-=140-or-dbp-=90)-(age-standardized-estimate). All projections generated during this study are included in this manuscript and its supplementary information file.
