# Supplementary material for: A glimpse into the future: modelling global prevalence of hypertension
Source: BMC Public Health. 2023 Oct 3;23:1906. doi: 10.1186/s12889-023-16662-z (PMC10546636; doi:10.1186/s12889-023-16662-z)
Supplement: Supplementary file 1 — Additional file 1: Table A1. Projected hypertension prevalence for 2040 (Male). Table A2. Projected hypertension prevalence for 2040 (Female). Table A3. Projected hypertension prevalence for 2040 (Both). Table A4. Changes in hypertension prevalence cluster patterns. Fig. A1a. A1h. Plot of actual data (dotted) and fitted curve (95% CI) for hypertension prevalence (Male). Fig. A2a. A2h. Plot of actual data (dotted) and fitted curve (95% CI) for hypertension prevalence (Female). Fig. A3a. A3h. Plot of actual data (dotted) and fitted curve (95% CI) for hypertension prevalence (Both). Fig. A4a. Plot of actual data and fitted curve (95% CI) for hypertension prevalence (Income groups). Fig. A4b. Plot of actual data and fitted curve (95% CI) for hypertension prevalence (Regions). [file 12889_2023_16662_MOESM1_ESM.pdf]

Supplementary material

A glimpse into the future: modelling global prevalence of hypertension

Contents

Table A1. Projected hypertension prevalence for 2040 (Male)	2
Table A2. Projected hypertension prevalence for 2040 (Female)	5
Table A3. Projected hypertension prevalence for 2040 (Both)	8
Fig. A1a – A1h. Plot of actual data (dotted) and fitted curve (95% CI) for hypertension prevalence (Male)	12
Fig. A2a – A2h. Plot of actual data (dotted) and fitted curve (95% CI) for hypertension prevalence (Female)	20
Fig. A3a – A3h. Plot of actual data (dotted) and fitted curve (95% CI) for hypertension prevalence (Both)	28
Fig. A4a. Plot of actual data and fitted curve (95% CI) for hypertension prevalence (Income groups)	36
Fig. A4b. Plot of actual data and fitted curve (95% CI) for hypertension prevalence (Regions)	37
Table A4. Changes in hypertension prevalence cluster patterns	38

Table A1. Projected hypertension prevalence for 2040 (Male).

No.	Country	2015[a]	2040[a]	Δ%[b]	L[c]	U[c]
1	Croatia	38.4	41.1	7.1	36.8	45.4
2	Trinidad and Tobago	27.6	40.8	47.8	40.5	41.1
3	Uganda	26.7	37.5	40.3	37.3	37.7
4	Bosnia and Herzegovina	34	37.1	9.1	33.7	40.3
5	Moldova	33.6	34.8	3.6	31.1	38.5
6	Pakistan	31.5	34.6	9.7	33.3	35.7
7	Romania	34.7	33.1	-4.7	28.9	37.1
8	Nepal	29.7	33.0	11.0	31.6	34.4
9	Burundi	27.3	32.2	17.9	30.4	33.8
10	Kazakhstan	30.4	31.8	4.8	28.7	34.7
11	Ethiopia	28.8	31.8	10.6	30.2	33.3
12	Saint Lucia	29.9	31.7	6.0	29.3	34.1
13	Slovenia	35.8	31.6	-11.7	27.9	35.4
14	Yemen	29.9	31.5	5.2	28.4	34.2
15	Lithuania	36.1	31.4	-13.1	26.3	37.0
16	Mali	31.2	31.2	0.1	28.9	33.3
17	Somalia	33.5	31.1	-7.1	27.4	34.7
18	Afghanistan	30.4	31.1	2.3	29.0	33.3
19	Sudan	30.6	30.8	0.8	28.7	32.8
20	Malawi	27.8	30.7	10.4	29.0	32.4
21	Albania	33	30.7	-7.1	28.4	33.2
22	Central African Republic	31.4	30.5	-2.9	28.7	32.3
23	Chad	31.6	30.4	-3.9	27.9	32.8
24	Vanuatu	24.2	30.3	25.2	30.1	30.4
25	Eritrea	28.2	30.2	7.2	29.1	31.5
26	Micronesia	26.6	29.8	12.0	27.9	31.6
27	Burkina Faso	31.3	29.8	-4.9	27.4	32.0
28	Comoros	27.4	29.4	7.4	28.1	30.8
29	Bhutan	28.5	29.4	3.2	28.3	30.5
30	Papua New Guinea	25.1	29.2	16.4	28.3	30.1
31	Macedonia	32.7	29.2	-10.8	26.9	31.5
32	Niger	31.3	29.1	-7.0	26.9	31.3
33	Cambodia	26.3	29.0	10.4	27.1	30.8
34	Poland	34.6	29.0	-16.2	25.6	33.1
35	Montenegro	34.4	28.9	-16.1	24.5	33.4
36	Tajikistan	26.4	28.8	9.1	27.1	30.7
37	Slovakia	34.3	28.7	-16.3	26.4	31.2
38	Antigua and Barbuda	26.4	28.6	8.3	25.8	31.4
39	Tanzania	26.6	28.5	7.1	27.5	29.4
40	Bulgaria	33.6	28.4	-15.6	24.1	33.1
41	Czechia	34.4	28.2	-17.9	24.6	32.5
42	Hungary	36.1	28.2	-21.8	26.0	30.8
43	Estonia	34.3	28.2	-17.8	24.9	32.0
44	Myanmar	24.9	28.1	12.9	27.9	28.3
45	India	26.6	28.1	5.6	27.3	28.9
46	Equatorial Guinea	29.2	28.1	-3.9	25.2	31.1
47	Congo, the Democratic Republic of the	29.3	28.0	-4.4	26.1	30.1
48	Guinea	29	27.9	-3.8	26.3	29.4
49	Viet Nam	25	27.8	11.2	26.7	28.8
50	Kenya	26.5	27.6	4.2	25.6	29.6
51	Ukraine	32.3	27.5	-14.9	24.9	30.3
52	Angola	29.6	27.3	-7.8	25.2	29.1
53	Kyrgyzstan	27.4	27.3	-0.5	26.2	28.3
54	Grenada	26.8	27.1	1.1	25.4	28.9
55	Russian Federation	32.6	27.1	-17.0	24.5	30.9
56	Saint Kitts and Nevis	27.9	27.0	-3.1	24.3	29.8
57	Uzbekistan	26.5	27.0	1.9	24.6	29.4
58	Niue	26.1	26.9	3.0	26.0	27.7

No.	Country	2015[a]	2040[a]	Δ%[b]	L[c]	U[c]
59	Mozambique	28.1	26.9	-4.4	24.6	29.0
60	Madagascar	27.8	26.9	-3.3	25.2	28.6
61	Guinea-Bissau	29.7	26.7	-10.2	24.7	28.5
62	Maldives	26.9	26.7	-0.9	24.6	28.7
63	Timor-Leste	26.9	26.6	-1.0	24.2	29.0
64	Armenia	27.8	26.6	-4.4	24.2	29.2
65	Mongolia	32.3	26.3	-18.7	23.8	29.1
66	Serbia	33.8	25.8	-23.6	21.7	29.8
67	Belarus	33	25.7	-22.2	23.9	28.4
68	Turkmenistan	26.6	25.6	-3.7	24.3	27.0
69	Rwanda	25.2	25.6	1.6	22.2	28.8
70	Botswana	29.3	25.2	-13.9	19.5	31.5
71	Barbados	27	25.2	-6.8	21.2	28.9
72	Eswatini	28.1	25.2	-10.4	22.2	28.0
73	Tuvalu	26.1	25.1	-3.7	23.7	26.5
74	Lesotho	26.1	24.7	-5.2	23.0	26.7
75	Sierra Leone	29.4	24.5	-16.5	22.0	27.7
76	Azerbaijan	25.8	24.5	-4.9	23.3	25.8
77	Thailand	24.2	24.3	0.5	22.4	26.3
78	Sri Lanka	23	24.3	5.5	22.4	26.2
79	Kiribati	24	24.2	0.9	23.0	25.4
80	Mauritania	31.8	24.2	-24.0	19.4	28.7
81	Togo	28.3	24.1	-14.7	21.8	26.5
82	Mauritius	26.5	24.1	-9.0	22.4	25.8
83	Senegal	29.7	24.1	-19.0	21.1	27.6
84	Morocco	26.2	23.8	-9.2	20.0	39.1
85	Liberia	28.2	23.6	-16.3	19.7	27.7
86	Namibia	28.2	23.5	-16.6	22.0	25.2
87	Zimbabwe	26.9	23.5	-12.7	21.8	25.3
88	Gambia	29.6	23.2	-21.7	20.0	26.2
89	Lao People's Democratic Republic	24.5	23.1	-5.7	21.3	24.9
90	Congo	27.5	22.9	-16.6	20.0	26.2
91	Argentina	27.6	22.7	-17.6	22.4	23.2
92	Philippines	24.1	22.7	-5.8	21.2	24.2
93	Cabo Verde	30.5	22.7	-25.7	19.0	26.8
94	Cote D'ivoire	27.8	22.5	-19.2	19.4	26.1
95	Saint Vincent and the Grenadines	25.9	22.4	-13.3	20.6	24.2
96	Egypt	24.8	22.4	-9.7	20.5	24.3
97	South Africa	27.4	22.2	-18.8	20.7	24.1
98	Haiti	25.2	22.2	-12.1	20.7	23.7
99	Bangladesh	24.5	22.0	-10.4	19.6	24.3
100	Palau	25.7	21.9	-15.0	20.7	23.1
101	Solomon Islands	20.4	21.4	5.1	19.8	23.0
102	Guatemala	22	21.4	-2.8	21.2	21.5
103	Iraq	25.6	21.4	-16.6	19.4	23.4
104	Seychelles	26.4	21.3	-19.5	18.7	24.1
105	Indonesia	24.3	21.2	-12.6	19.1	23.3
106	Jamaica	24.5	21.1	-14.0	18.3	23.8
107	Honduras	22.6	21.0	-6.9	19.5	22.7
108	Marshall Islands	23.8	20.9	-12.2	19.0	23.1
109	Djibouti	28	20.6	-26.3	18.6	23.0
110	Fiji	22.4	20.5	-8.4	18.1	23.1
111	Cook Islands	24.9	20.2	-18.9	18.9	21.4
112	Belize	24.4	20.1	-17.6	17.9	22.5
113	Brazil	26.7	19.8	-26.0	18.0	21.7
114	Benin	27.1	19.7	-27.3	16.5	23.5
115	Syrian Arab Republic	25.1	19.6	-22.0	17.1	22.1
116	Chile	25.4	19.5	-23.3	18.6	20.4
117	Nauru	23.8	19.3	-19.0	19.1	19.5

No.	Country	2015[a]	2040[a]	Δ%[b]	L[c]	U[c]
118	Dominica	25.7	19.3	-25.1	17.2	21.4
119	Suriname	24.6	19.2	-21.9	15.6	22.9
120	Panama	22.3	19.1	-14.5	17.9	20.1
121	Algeria	25.4	19.1	-24.9	16.6	21.6
122	Libya	25.2	19.1	-24.3	13.8	25.1
123	Mexico	22.3	18.8	-15.9	16.9	20.8
124	Portugal	29.3	18.6	-36.4	15.0	22.6
125	Malaysia	25.3	18.5	-26.7	15.0	22.3
126	China	21.5	18.5	-13.8	15.9	21.4
127	Nicaragua	21.6	18.3	-15.2	16.3	20.8
128	Bahamas	25.2	18.3	-27.5	16.5	20.3
129	Sao Tome and Principe	26.9	18.2	-32.4	15.5	21.1
130	Korea (North)	19.1	18.1	-5.3	18.0	18.2
131	Oman	25.7	18.0	-29.8	14.9	21.4
132	Tunisia	23.8	17.9	-24.7	16.9	18.9
133	Ecuador	19.8	17.8	-9.9	15.7	20.1
134	Ghana	24.6	17.2	-30.2	14.7	19.8
135	Jordan	23.1	17.1	-25.9	15.8	18.7
136	Belgium	22.4	17.0	-24.2	16.4	17.7
137	Dominican Republic	23.8	16.8	-29.3	12.7	21.4
138	Saudi Arabia	24.7	16.8	-32.0	13.6	20.1
139	Cameroon	24.9	16.7	-33.0	13.8	19.7
140	Austria	25.2	16.7	-33.8	15.7	17.8
141	Luxembourg	27.8	16.6	-40.2	13.6	21.8
142	El Salvador	20.4	16.6	-18.8	15.3	17.9
143	Colombia	21.5	16.5	-23.3	15.5	17.5
144	Bolivia	19.7	16.4	-16.7	13.3	20.2
145	Iceland	26.2	16.3	-37.9	13.0	20.3
146	Brunei Darussalam	22	16.3	-26.1	14.4	18.2
147	Finland	24	16.2	-32.4	15.4	17.4
148	Costa Rica	21	16.0	-23.6	13.9	18.0
149	Japan	22.5	16.0	-29.1	15.0	17.0
150	Greece	22.8	15.8	-30.5	14.3	17.5
151	Switzerland	22.3	15.4	-31.1	11.9	19.2
152	Kuwait	25.5	15.2	-40.3	11.4	21.3
153	Uruguay	24.7	14.8	-39.9	13.8	16.2
154	Cyprus	23.9	14.8	-38.1	11.6	18.8
155	Germany	24.3	14.7	-39.6	13.5	16.0
156	Bahrain	22.5	14.6	-35.0	13.6	15.8
157	Venezuela	21.5	14.5	-32.4	13.4	15.9
158	Andorra	23.2	14.4	-37.9	13.2	16.0
159	Cuba	20.9	13.9	-33.4	10.8	17.6
160	France	27.7	13.7	-50.6	11.1	16.5
161	Qatar	23	13.5	-41.3	12.8	14.2
162	Malta	24.3	13.3	-45.5	11.5	15.1
163	Nigeria	22.7	13.0	-42.7	9.0	17.8
164	Norway	24.6	12.9	-47.6	10.9	14.9
165	United States	15.3	12.8	-16.6	11.9	13.8
166	Iran (Islamic Republic of)	20.4	12.7	-37.8	10.8	14.8
167	Italy	25.2	12.5	-50.5	10.2	15.3
168	New Zealand	19.3	12.2	-36.9	10.5	14.1
169	Israel	20.6	12.1	-41.1	11.3	13.1
170	Turkey	20.3	12.0	-40.7	9.0	15.6
171	Peru	16.1	12.0	-25.5	11.6	12.4
172	Sweden	24.1	10.9	-54.9	10.0	11.8
173	Denmark	26.5	10.5	-60.3	6.6	15.0
174	Netherlands	23.1	10.1	-56.4	8.7	11.6
175	Spain	23.5	9.7	-58.7	7.3	12.5
176	Ireland	22.8	8.4	-63.0	5.1	13.7

No.	Country	2015[a]	2040[a]	Δ%[b]	L[c]	U[c]
177	Singapore	17.8	7.9	-55.9	5.8	10.5
178	Australia	18	7.6	-57.6	6.6	8.9
179	Canada	15.6	7.3	-53.3	6.6	8.0
180	United Kingdom	17.9	5.5	-69.0	4.1	7.4

[a] Raised BP (SBP>=140 OR DBP>=90) (%, age-standardised estimate), 18+ years.

[b] Percentage change in hypertension prevalence from 2015 to 2040.

[c] Lower and upper confidence intervals (95%) for the 2040 forecast.0

Table A2. Projected hypertension prevalence for 2040 (Female).

No.	Country	2015[a]	2040[a]	Δ%[b]	L[c]	U[c]
1	Niger	35.8	38.7	8.2	38.4	39.0
2	Chad	33.8	38.6	14.1	35.9	40.8
3	Ethiopia	31.7	38.1	20.2	37.3	38.8
4	Burundi	31.1	37.3	19.9	36.3	38.3
5	Mali	33.6	34.9	4.0	32.8	36.9
6	Burkina Faso	33.2	34.7	4.6	32.7	36.8
7	Malawi	29.6	34.1	15.2	32.6	35.6
8	Afghanistan	30.7	33.5	9.1	32.8	34.2
9	Uganda	27.7	33.0	19.1	32.5	33.5
10	Eritrea	29.5	32.8	11.1	31.9	33.6
11	Nepal	29.5	32.7	11.0	31.6	33.8
12	Guinea	31.4	32.6	3.7	30.8	34.4
13	Lesotho	30.8	32.5	5.4	31.1	33.9
14	Somalia	32.2	32.2	0.0	30.2	34.0
15	Eswatini	30.9	32.1	3.9	29.1	35.2
16	Papua New Guinea	25.8	32.0	24.2	31.1	33.0
17	Mozambique	29.7	31.8	7.2	29.4	34.0
18	Comoros	28.2	31.1	10.4	28.9	33.2
19	Sudan	29.6	31.1	5.1	29.2	33.1
20	Pakistan	29.5	30.9	4.9	30.4	31.5
21	Yemen	31.2	30.8	-1.3	28.9	32.7
22	Central African Republic	30.8	30.6	-0.6	29.2	31.9
23	Timor-Leste	28.1	30.6	8.8	29.3	31.8
24	Rwanda	27.9	30.2	8.2	28.0	32.3
25	Tanzania	27.7	29.2	5.5	27.9	30.5
26	Madagascar	28.2	29.1	3.2	28.0	30.2
27	Kenya	26.7	28.9	8.1	27.5	30.2
28	Trinidad and Tobago	23.9	28.4	18.8	26.8	29.7
29	Guinea-Bissau	30.7	28.3	-7.7	25.4	31.0
30	Senegal	30.4	28.2	-7.3	26.1	30.5
31	Bhutan	27.6	28.1	1.7	26.5	29.5
32	Zimbabwe	29.2	28.0	-4.3	25.6	30.1
33	Cambodia	25.5	27.9	9.3	24.9	30.6
34	Togo	29.2	27.7	-5.2	25.7	29.6
35	Solomon Islands	23.6	27.5	16.5	26.7	28.2
36	Micronesia	23.2	27.5	18.4	25.9	29.0
37	Tajikistan	25.7	27.4	6.7	25.4	29.3
38	Saint Lucia	24.4	27.3	12.0	25.6	29.0
39	Sierra Leone	31	27.2	-12.2	24.3	30.3
40	Equatorial Guinea	27.7	27.0	-2.4	25.2	28.9
41	Vanuatu	24.1	26.8	11.3	25.7	28.2
42	Angola	29.6	26.8	-9.6	24.5	29.3
43	Tonga	21.8	26.6	22.1	24.7	28.5
44	Congo, the Democratic Republic of the	27.6	26.5	-4.1	24.9	28.2
45	Kyrgyzstan	25.7	25.8	0.5	24.5	27.2
46	India	24.7	25.5	3.2	24.7	26.2
47	Bosnia and Herzegovina	27.6	25.4	-8.0	22.4	28.6

No.	Country	2015[a]	2040[a]	Δ%[b]	L[c]	U[c]
48	Liberia	28.3	25.4	-10.3	21.3	30.1
49	Zambia	26.5	25.0	-5.6	22.6	27.4
50	Mauritania	31.4	25.0	-20.5	20.8	28.8
51	Gambia	28.8	24.7	-14.3	21.5	27.4
52	Sri Lanka	21.6	24.6	14.1	23.5	25.7
53	Myanmar	24.2	24.4	0.8	23.0	25.9
54	Uzbekistan	24.4	24.3	-0.4	22.5	26.3
55	Benin	28.1	24.3	-13.6	21.2	27.9
56	Samoa	21	24.0	14.4	21.2	26.8
57	Lao People's Democratic Republic	24.9	24.0	-3.6	22.1	25.8
58	Namibia	28.4	24.0	-15.5	22.0	25.9
59	Botswana	29.5	23.8	-19.3	19.4	28.7
60	Guyana	21.5	23.3	8.5	21.4	25.4
61	Moldova	26.2	23.0	-12.3	20.4	25.7
62	Maldives	21.9	22.9	4.7	20.1	25.6
63	Bangladesh	24.9	22.9	-8.1	20.1	25.6
64	Niue	22.1	22.8	3.2	22.0	23.5
65	Cote D'ivoire	26.8	22.8	-15.0	19.7	26.1
66	Georgia	23.8	22.5	-5.4	21.1	24.1
67	Viet Nam	21.6	22.5	4.2	21.4	23.7
68	Saint Kitts and Nevis	22.7	22.4	-1.3	21.2	23.7
69	Croatia	26.3	22.3	-15.4	19.5	25.3
70	Mauritius	23.4	21.7	-7.2	20.5	23.1
71	Romania	25.2	21.5	-14.8	19.7	23.3
72	Antigua and Barbuda	20.4	21.4	4.9	20.1	22.8
73	Turkmenistan	24.1	21.3	-11.6	20.3	22.3
74	Cabo Verde	28.1	21.1	-25.0	16.4	25.8
75	Philippines	21	20.5	-2.6	18.8	22.4
76	Grenada	21.6	20.4	-5.8	18.5	22.1
77	Slovenia	24.9	20.1	-19.4	17.2	23.1
78	Egypt	25	20.0	-20.0	18.7	21.3
79	Kazakhstan	23.9	19.9	-16.6	18.5	21.4
80	Thailand	20.3	19.9	-2.2	18.6	21.1
81	Barbados	21.7	19.8	-8.5	17.5	22.2
82	Azerbaijan	23	19.7	-14.2	19.3	20.2
83	Indonesia	23.1	19.4	-16.0	17.2	21.6
84	South Africa	26.1	19.4	-25.7	18.2	20.8
85	Congo	24.9	19.2	-23.1	16.7	21.4
86	Tuvalu	21.2	19.1	-9.8	18.2	20.1
87	Poland	23	19.0	-17.4	18.6	19.5
88	Macedonia	24.1	18.7	-22.3	17.1	20.7
89	Cameroon	24.6	18.6	-24.5	15.6	21.6
90	Armenia	22.9	18.3	-20.3	17.5	19.1
91	Belize	21	18.2	-13.1	17.0	19.6
92	Nigeria	25	18.1	-27.7	14.9	21.7
93	Djibouti	25.5	18.0	-29.2	16.5	19.6
94	Serbia	25.2	18.0	-28.4	15.9	20.3
95	Haiti	23.7	18.0	-23.9	15.3	20.8
96	Albania	25	18.0	-28.2	16.4	19.5
97	Lithuania	23.1	17.8	-23.0	17.1	18.7
98	Fiji	20.7	17.6	-14.8	15.5	20.1
99	Morocco	25.8	17.4	-32.5	14.4	20.6
100	Mongolia	25.6	17.4	-32.2	15.2	19.6
101	Slovakia	22.8	17.2	-24.6	14.8	19.7
102	Guatemala	20.4	17.2	-15.8	16.0	18.4
103	Czechia	21.2	17.1	-19.5	16.4	17.8
104	Estonia	20.9	16.9	-18.9	16.7	17.3
105	Ghana	22.8	16.9	-25.8	13.9	19.6
106	Sao Tome and Principe	24.6	16.8	-31.9	13.7	20.1

No.	Country	2015[a]	2040[a]	Δ%[b]	L[c]	U[c]
107	Syrian Arab Republic	23.8	16.5	-30.5	15.4	17.9
108	Iraq	24.4	16.5	-32.5	14.2	19.2
109	Honduras	20.2	16.4	-18.8	15.2	17.7
110	Montenegro	23.8	16.4	-31.1	14.3	18.6
111	Hungary	24	16.1	-32.8	14.7	17.7
112	Ukraine	22.3	16.1	-27.7	15.4	17.0
113	Saint Vincent and the Grenadines	20.7	16.1	-22.4	14.7	17.4
114	Bulgaria	23	15.9	-30.7	13.9	18.4
115	Paraguay	21.3	15.8	-26.0	14.4	17.4
116	Seychelles	20.2	15.6	-22.5	13.0	18.6
117	Jamaica	19.2	15.3	-20.5	13.1	17.6
118	Nicaragua	19.9	15.2	-23.9	13.4	17.2
119	Cook Islands	19.5	14.8	-23.9	13.9	15.8
120	Gabon	23	14.8	-35.7	10.5	19.7
121	Malaysia	20.8	14.8	-29.1	12.2	17.4
122	Marshall Islands	18.6	14.7	-20.7	12.3	17.5
123	Libya	22.2	14.4	-35.3	12.2	17.3
124	Palau	20	14.4	-28.2	13.2	15.6
125	Russian Federation	22.3	14.3	-35.9	12.9	16.0
126	Suriname	19.9	14.2	-28.7	12.1	16.7
127	Tunisia	22.5	14.1	-37.5	12.4	15.9
128	Latvia	22.9	14.0	-38.7	11.4	16.9
129	Algeria	24.6	13.9	-43.3	10.9	16.8
130	Korea (North)	16.9	13.9	-18.0	12.0	15.8
131	Belarus	21.6	13.6	-36.8	12.5	15.3
132	Dominica	19.4	12.9	-33.3	10.9	15.3
133	Lebanon	17.8	12.5	-29.7	11.4	14.3
134	Panama	17.3	12.4	-28.1	11.1	13.8
135	Nauru	17.2	12.2	-29.1	11.9	12.6
136	Mexico	17.3	11.9	-31.4	11.3	12.4
137	El Salvador	17.2	11.7	-31.8	10.7	12.8
138	Argentina	17.6	11.3	-35.9	11.7	17.6
139	Brunei Darussalam	15.8	11.0	-30.7	9.0	13.1
140	Bahamas	16.8	10.9	-35.2	10.5	11.4
141	Jordan	18.8	10.8	-42.4	9.8	11.9
142	Brazil	19.9	10.8	-45.8	9.2	12.5
143	Cuba	16.9	10.8	-36.4	9.7	12.1
144	Ecuador	16	10.7	-32.8	9.4	12.2
145	Bahrain	19.1	10.7	-43.8	10.5	11.0
146	United Arab Emirates	18.3	10.6	-42.0	9.4	11.8
147	Colombia	16.9	10.6	-37.2	9.7	11.6
148	Dominican Republic	19.1	10.6	-44.6	7.9	13.5
149	Austria	16.8	10.6	-37.2	10.0	11.1
150	Chile	16.5	10.5	-36.3	10.1	10.9
151	Bolivia	16.1	10.4	-35.1	8.7	12.6
152	Venezuela	15.7	9.7	-38.3	9.0	10.5
153	Kuwait	20.3	9.6	-52.6	8.5	10.9
154	Portugal	19.6	9.5	-51.4	6.5	12.9
155	Greece	15.4	9.5	-38.3	8.9	10.1
156	New Zealand	13.3	9.5	-28.6	9.3	9.7
157	Costa Rica	16.3	9.4	-42.5	7.9	11.0
158	Germany	15.5	9.4	-39.6	8.9	9.9
159	Cyprus	15.5	9.3	-40.2	8.0	10.9
160	Belgium	12.6	9.2	-27.3	9.1	9.3
161	Iran (Islamic Republic of)	18.9	8.7	-54.1	6.5	11.0
162	United States	10.5	8.7	-17.6	8.5	8.8
163	Qatar	19.1	8.6	-54.7	8.1	9.2
164	Turkey	20.1	8.6	-57.0	6.1	12.5
165	Uruguay	16.8	8.6	-48.9	8.3	8.9

No.	Country	2015[a]	2040[a]	Δ%[b]	L[c]	U[c]
166	Andorra	14.2	8.5	-40.4	8.0	9.1
167	Switzerland	13.7	8.3	-39.2	6.8	10.3
168	Finland	14.7	7.9	-46.6	7.2	8.7
169	Italy	17.1	7.8	-54.4	6.6	9.1
170	France	16.4	7.7	-52.7	7.0	8.5
171	Luxembourg	15.9	7.5	-52.7	6.4	8.8
172	Norway	14.7	7.5	-49.2	6.9	8.1
173	Ireland	16.5	7.3	-55.8	5.6	9.1
174	Japan	12.6	7.3	-42.3	6.8	7.9
175	Spain	14.7	6.9	-52.8	6.2	7.8
176	Iceland	13	6.8	-47.4	6.0	7.9
177	Australia	12.3	6.7	-45.2	6.4	7.1
178	Israel	12.8	6.5	-49.3	6.2	6.9
179	Malta	14.3	6.2	-56.7	5.9	6.5
180	Sweden	14.4	6.1	-57.6	5.6	6.8
181	Canada	10.8	5.9	-45.7	5.5	6.2
182	Denmark	14.8	5.2	-64.8	3.8	6.7
183	United Kingdom	12.4	4.9	-60.1	4.2	5.9
184	Netherlands	14.3	4.4	-69.3	3.5	5.3
185	Peru	11.2	4.4	-60.8	3.4	5.5
186	Singapore	11.3	3.9	-65.7	2.8	5.3

[a] Raised BP (SBP>=140 OR DBP>=90) (%, age-standardised estimate), 18+ years.

[b] Percentage change in hypertension prevalence from 2015 to 2040.

[c] Lower and upper confidence intervals (95%) for the 2040 forecast.

Table A3. Projected hypertension prevalence for 2040 (Both).

No.	Country	2015[a]	2040[a]	Δ%[b]	L[c]	U[c]
1	Chad	32.9	34.9	6.2	32.6	37.2
2	Burundi	29.2	34.9	19.6	33.6	36.1
3	Niger	33.4	34.0	1.8	32.4	35.5
4	Mali	32.6	33.2	1.7	31.4	34.8
5	Malawi	28.9	32.9	13.7	31.6	34.1
6	Pakistan	30.5	32.9	7.7	31.8	33.9
7	Uganda	27.3	32.8	20.1	31.9	33.6
8	Burkina Faso	32.6	32.7	0.3	30.2	35.1
9	Afghanistan	30.6	32.4	5.8	31.1	33.7
10	Nepal	29.4	32.2	9.4	30.8	33.4
11	Somalia	32.9	31.9	-3.1	29.5	34.3
12	Eritrea	29.1	31.6	8.7	33.0	50.0
13	Yemen	30.7	31.6	2.9	28.3	34.9
14	Croatia	32.4	31.5	-2.7	28.1	35.5
15	Bosnia and Herzegovina	30.8	31.3	1.8	26.5	35.8
16	Sudan	30.2	31.1	3.0	29.1	33.0
17	Central African Republic	31.2	30.7	-1.6	29.1	32.3
18	Papua New Guinea	25.6	30.6	19.6	29.6	31.5
19	Trinidad and Tobago	25.8	30.5	18.1	29.1	31.8
20	Comoros	27.9	30.5	9.2	29.0	31.9
21	Guinea	30.3	30.3	0.1	28.7	32.0
22	Mozambique	29.1	30.0	3.0	28.0	31.9
23	Saint Lucia	27.1	29.6	9.1	27.3	31.6
24	Eswatini	29.8	29.4	-1.3	25.6	33.1
25	Lesotho	29	29.4	1.3	28.1	30.8
26	Tanzania	27.3	29.0	6.2	28.1	29.9
27	Timor-Leste	27.6	28.8	4.4	27.2	30.5
28	Micronesia	25	28.8	15.3	27.3	30.3
29	Moldova	29.8	28.7	-3.8	25.6	32.0
30	Cambodia	26.1	28.7	9.8	26.1	31.0

No.	Country	2015[a]	2040[a]	Δ%[b]	L[c]	U[c]
31	Bhutan	28.1	28.6	1.9	27.2	30.0
32	Tajikistan	26.1	28.5	9.1	26.4	30.6
33	Kenya	26.7	28.4	6.4	27.0	29.8
34	Rwanda	26.7	28.2	5.6	25.2	30.9
35	Madagascar	28.1	28.1	0.1	26.9	29.5
36	Tonga	23.7	28.1	18.5	26.1	30.0
37	Guinea-Bissau	30.3	27.7	-8.7	25.6	29.7
38	Romania	30	27.5	-8.3	24.7	30.1
39	Equatorial Guinea	28.4	27.4	-3.4	24.7	30.3
40	Congo, the Democratic Republic of the	28.5	27.4	-3.8	25.8	29.2
41	Angola	29.7	27.0	-9.1	24.9	29.2
42	Georgia	26.3	26.9	2.1	24.6	29.4
43	India	25.8	26.8	4.0	26.0	27.6
44	Senegal	30.2	26.7	-11.5	24.4	29.1
45	Vanuatu	24.2	26.7	10.3	25.1	28.4
46	Kyrgyzstan	26.7	26.7	0.0	25.4	28.0
47	Slovenia	30.5	26.3	-13.8	23.1	29.8
48	Togo	28.9	26.2	-9.4	24.0	28.3
49	Sierra Leone	30.3	26.1	-13.9	23.1	29.1
50	Zimbabwe	28.2	26.0	-7.6	24.3	28.0
51	Uzbekistan	25.6	25.7	0.6	23.8	27.6
52	Zambia	27.1	25.5	-5.9	23.2	27.7
53	Kazakhstan	27.1	25.4	-6.3	23.1	27.5
54	Myanmar	24.6	25.3	3.0	24.4	26.3
55	Viet Nam	23.4	25.2	7.8	24.6	26.0
56	Lithuania	29.3	25.1	-14.4	24.3	26.3
57	Niue	24.2	24.9	2.9	24.2	25.6
58	Antigua and Barbuda	23.4	24.8	6.1	23.2	26.8
59	Botswana	29.6	24.8	-16.3	19.1	31.0
60	Maldives	24.4	24.7	1.4	22.8	26.9
61	Saint Kitts and Nevis	25.3	24.7	-2.4	23.5	26.0
62	Albania	29	24.7	-14.9	23.0	26.1
63	Mauritania	31.7	24.6	-22.3	20.8	28.7
64	Liberia	28.3	24.6	-13.1	21.1	28.4
65	Latvia	29.4	24.5	-16.7	20.9	28.7
66	Solomon Islands	22	24.5	11.3	23.4	25.7
67	Guyana	23.1	24.3	5.1	22.3	26.2
68	Gambia	29.1	24.1	-17.0	21.4	27.1
69	Namibia	28.5	24.0	-15.8	22.5	25.4
70	Macedonia	28.5	23.9	-16.1	21.6	26.4
71	Lao People's Democratic Republic	24.8	23.7	-4.4	22.0	25.5
72	Grenada	24.3	23.7	-2.6	22.3	25.0
73	Turkmenistan	25.4	23.3	-8.2	22.2	24.4
74	Slovakia	28.5	23.1	-19.0	20.7	25.7
75	Mauritius	25	22.9	-8.5	21.3	24.5
76	Barbados	24.4	22.8	-6.6	20.0	25.9
77	Poland	28.7	22.7	-21.0	20.3	25.7
78	Montenegro	29.1	22.6	-22.4	19.9	25.6
79	Cote D'ivoire	27.2	22.5	-17.3	19.6	25.3
80	Armenia	25.5	22.4	-12.1	20.4	24.4
81	Hungary	30	22.4	-25.4	20.6	24.4
82	Bangladesh	24.7	22.4	-9.4	20.0	25.1
83	Azerbaijan	24.5	22.2	-9.5	21.1	23.2
84	Brunei Darussalam	28.4	22.2	-21.9	19.4	25.7
85	Cabo Verde	29.5	22.2	-24.9	17.1	27.1
86	Thailand	22.3	22.1	-0.7	20.4	24.1
87	Serbia	29.5	22.0	-25.4	19.2	25.1
88	Benin	27.7	22.0	-20.6	18.4	25.7
89	Tuvalu	23.7	22.0	-7.2	20.8	23.1

No.	Country	2015[a]	2040[a]	Δ%[b]	L[c]	U[c]
90	Mongolia	29	22.0	-24.2	19.7	24.6
91	Czechia	27.9	21.8	-22.0	18.9	25.0
92	Philippines	22.6	21.6	-4.6	20.2	22.9
93	Kiribati	21.5	21.5	0.1	20.5	22.5
94	Estonia	27.4	21.4	-22.0	19.9	23.4
95	Egypt	25	21.3	-14.9	20.7	21.8
96	Ukraine	27.1	21.1	-22.0	19.7	23.1
97	Congo	26.2	21.1	-19.6	18.6	23.6
98	South Africa	26.9	20.7	-23.1	19.4	22.0
99	Indonesia	23.8	20.4	-14.3	18.5	22.3
100	Haiti	24.5	20.2	-17.4	17.9	22.4
101	Russian Federation	27.2	20.0	-26.5	18.2	22.5
102	Morocco	26.1	19.8	-24.3	17.8	21.8
103	Djibouti	26.8	19.7	-26.5	18.2	21.5
104	Ethiopia	30.3	19.3	-36.5	16.9	21.6
105	Belize	22.7	19.1	-15.7	17.6	20.8
106	Saint Vincent and the Grenadines	23.3	19.1	-18.1	17.7	20.4
107	Belarus	27.1	19.0	-29.9	17.2	21.3
108	Iraq	25.2	18.9	-24.9	16.9	21.1
109	Paraguay	24.6	18.8	-23.4	16.7	21.1
110	Seychelles	23.5	18.8	-20.1	16.4	21.1
111	Guatemala	21.2	18.7	-12.0	16.7	20.6
112	Honduras	21.4	18.6	-13.2	17.4	19.7
113	Jamaica	21.8	18.1	-17.0	15.8	20.3
114	Palau	22.9	18.1	-21.1	16.7	19.5
115	Syrian Arab Republic	24.5	18.1	-26.3	16.4	19.9
116	Gabon	25.5	18.0	-29.5	13.9	23.6
117	Marshall Islands	21.3	17.9	-16.2	16.2	19.6
118	Cook Islands	22.3	17.8	-20.4	16.8	18.6
119	Sao Tome and Principe	25.8	17.7	-31.4	14.9	20.8
120	Cameroon	24.8	17.6	-28.9	14.9	20.6
121	Suriname	22.4	17.6	-21.4	16.3	19.4
122	Argentina	22.6	17.5	-22.8	17.2	17.7
123	Algeria	25.1	17.4	-30.8	15.5	19.8
124	Oman	24.8	17.1	-31.0	13.2	21.7
125	Ghana	23.7	17.0	-28.2	14.5	19.7
126	Nicaragua	20.8	16.8	-19.0	15.2	18.4
127	Malaysia	22.9	16.4	-28.2	13.5	19.9
128	Korea (North)	18.2	16.3	-10.5	16.2	16.4
129	Tunisia	23.2	16.0	-30.9	14.5	17.5
130	Dominica	22.5	15.9	-29.1	14.1	18.2
131	Panama	19.9	15.9	-20.2	15.0	16.8
132	Nauru	20.5	15.5	-24.2	15.2	16.0
133	Brazil	23.3	15.5	-33.3	14.0	17.2
134	Lebanon	20.7	15.4	-25.6	12.1	19.4
135	Chile	20.9	15.3	-26.6	14.9	15.8
136	Nigeria	23.9	15.3	-35.9	11.4	19.7
137	China	19.2	15.3	-20.4	12.4	18.2
138	Saudi Arabia	23.3	14.8	-36.5	12.5	17.9
139	Mexico	19.7	14.3	-27.5	13.2	15.5
140	Bahamas	20.9	14.2	-32.2	13.2	15.2
141	Ecuador	17.9	14.1	-21.1	12.3	16.2
142	Portugal	24.4	14.0	-42.6	10.8	17.3
143	El Salvador	18.7	13.9	-25.6	13.0	14.9
144	Dominican Republic	21.5	13.9	-35.4	10.7	17.0
145	Jordan	21	13.9	-34.0	12.4	15.7
146	Colombia	19.2	13.7	-28.8	12.8	14.6
147	Brunei Darussalam	18.9	13.6	-28.1	11.8	15.6
148	Belgium	17.5	13.3	-23.9	13.1	13.6

No.	Country	2015[a]	2040[a]	Δ%[b]	L[c]	U[c]
149	Austria	21	13.1	-37.7	12.2	14.1
150	Bolivia	17.9	13.1	-27.0	11.1	15.3
151	United Arab Emirates	21.1	12.9	-38.9	11.2	15.3
152	Qatar	22.4	12.9	-42.5	11.2	14.5
153	Bahrain	21.4	12.9	-39.9	12.1	13.6
154	Costa Rica	18.7	12.6	-32.5	11.0	14.4
155	Kuwait	23.6	12.4	-47.3	9.5	16.4
156	Venezuela	18.6	12.3	-34.1	11.5	13.2
157	Luxembourg	21.9	12.2	-44.1	10.2	14.6
158	Greece	19.1	12.1	-36.6	11.2	13.1
159	Switzerland	18	12.1	-32.9	9.7	15.3
160	Cyprus	19.8	12.0	-39.5	9.3	15.5
161	Cuba	19	11.9	-37.1	9.6	14.5
162	Uruguay	20.7	11.9	-42.5	11.3	12.6
163	Japan	17.6	11.6	-34.0	10.9	12.4
164	United States	12.9	11.5	-10.7	11.5	11.6
165	Germany	19.9	11.5	-42.3	10.6	12.6
166	Iran (Islamic Republic of)	19.7	11.4	-41.9	9.9	13.2
167	New Zealand	16.2	11.3	-30.4	10.9	11.8
168	Andorra	18.7	11.2	-40.0	10.3	12.3
169	Fiji	21.7	11.1	-48.9	9.9	12.5
170	Finland	19.4	11.1	-42.9	9.9	12.5
171	Iceland	19.7	10.9	-44.4	9.7	12.3
172	Turkey	20.3	10.6	-48.0	7.8	13.6
173	France	22	10.3	-53.3	8.8	11.7
174	Norway	19.7	9.9	-49.9	9.3	10.5
175	Italy	21.2	9.7	-54.4	7.8	11.9
176	Malta	19.4	9.6	-50.5	8.9	10.3
177	Ireland	19.7	9.2	-53.4	7.2	11.5
178	Israel	16.6	8.9	-46.6	8.3	9.5
179	Spain	19.2	8.4	-56.1	7.0	10.3
180	Peru	13.7	8.3	-39.7	7.7	9.0
181	Denmark	20.6	7.8	-62.1	4.9	10.9
182	Sweden	19.3	7.5	-61.0	6.6	8.7
183	Australia	15.2	6.8	-55.1	6.2	7.5
184	Netherlands	18.7	6.6	-64.7	5.5	7.7
185	Singapore	14.6	6.1	-57.9	4.7	7.9
186	Canada	13.2	5.7	-56.7	5.1	6.3
187	United Kingdom	15.2	5.7	-62.7	4.8	6.7

[a] Raised BP (SBP>=140 OR DBP>=90) (%, age-standardised estimate), 18+ years.

[b] Percentage change in hypertension prevalence from 2015 to 2040.

[c] Lower and upper confidence intervals (95%) for the 2040 forecast.

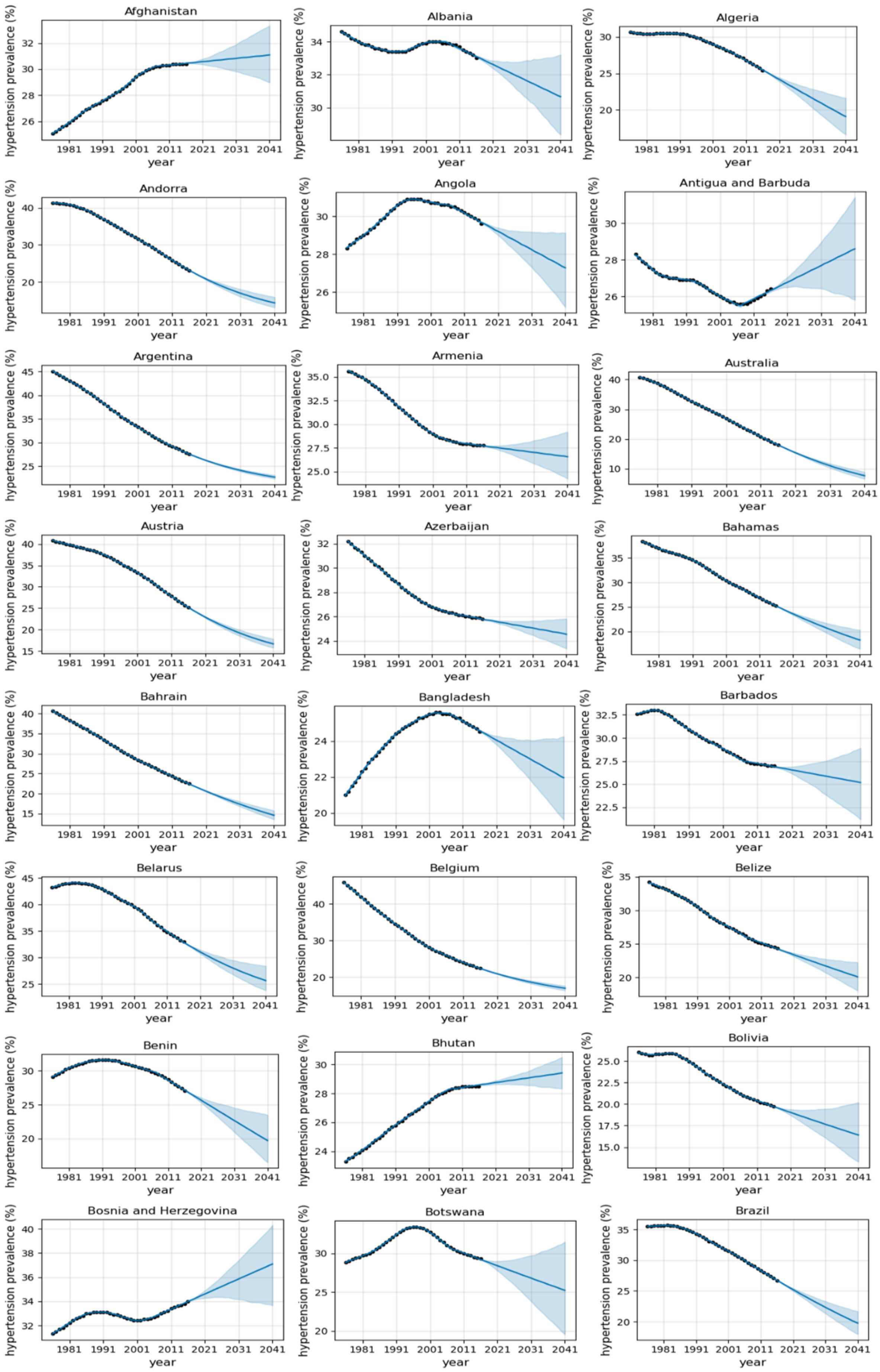

Fig. A1a. Plot of actual data (dotted) and fitted curve (95% CI) for hypertension prevalence (Male)

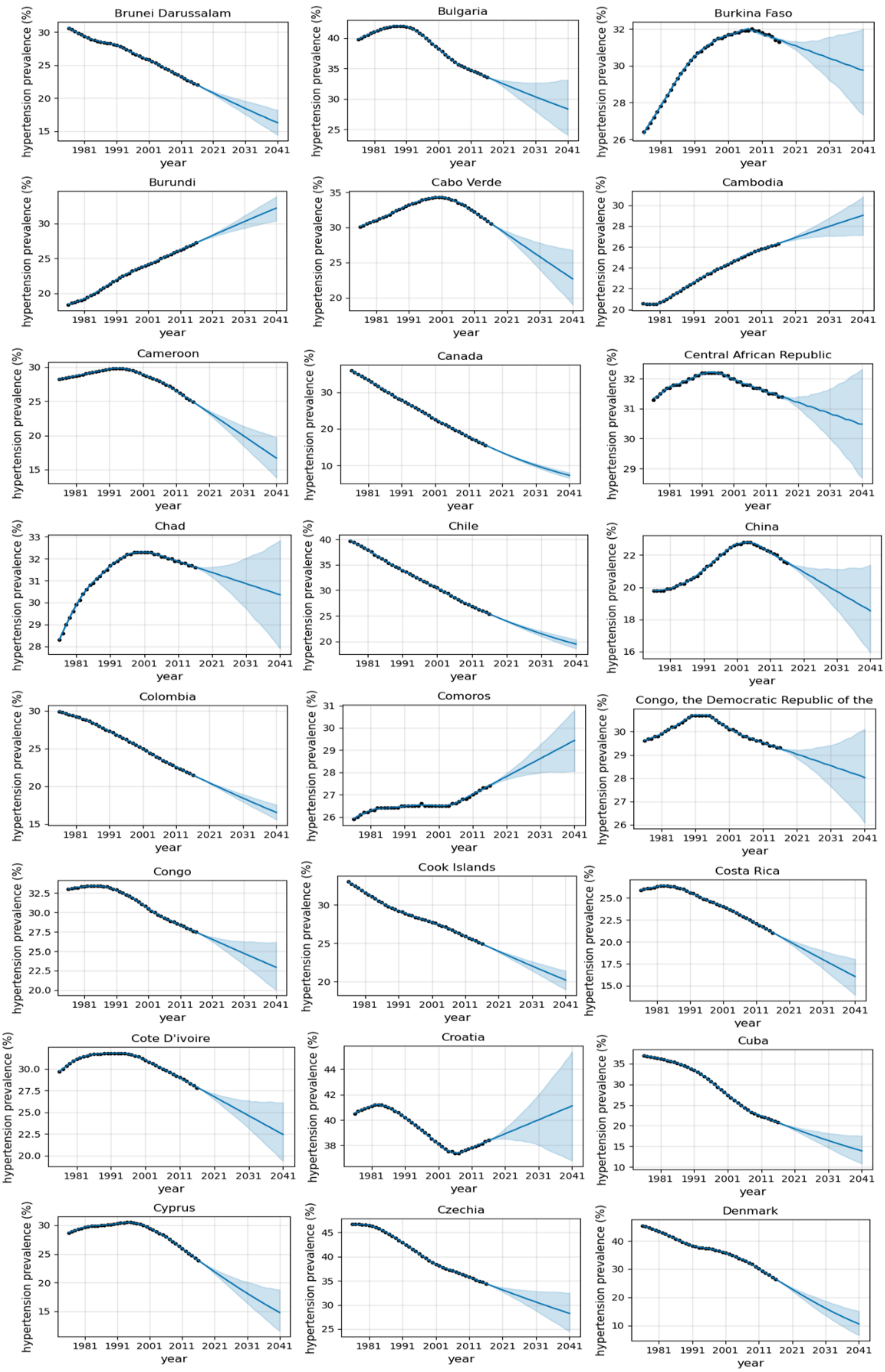

Fig. A1b. Plot of actual data (dotted) and fitted curve (95% CI) for hypertension prevalence (Male)

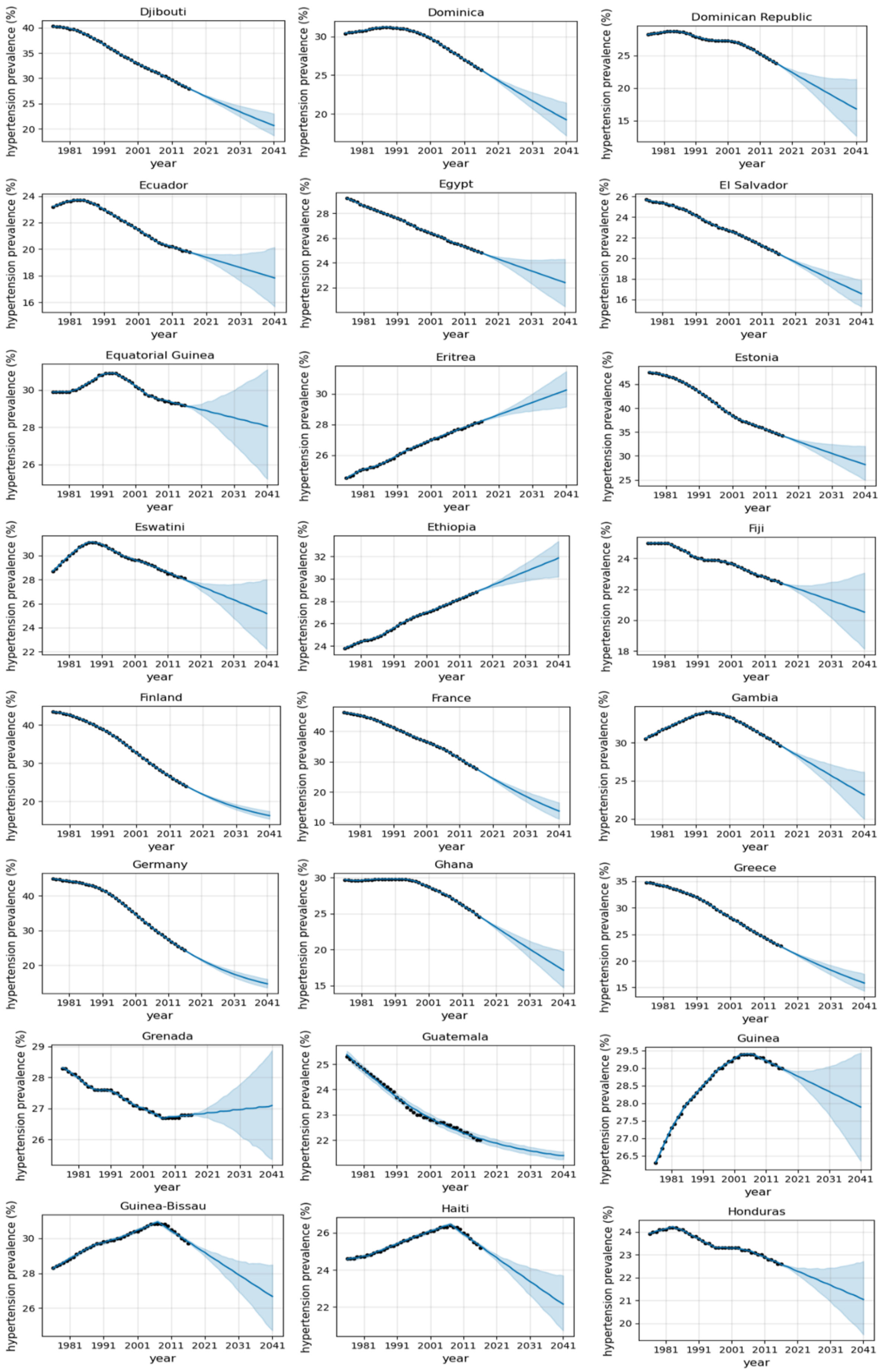

Fig. A1c. Plot of actual data (dotted) and fitted curve (95% CI) for hypertension prevalence (Male)

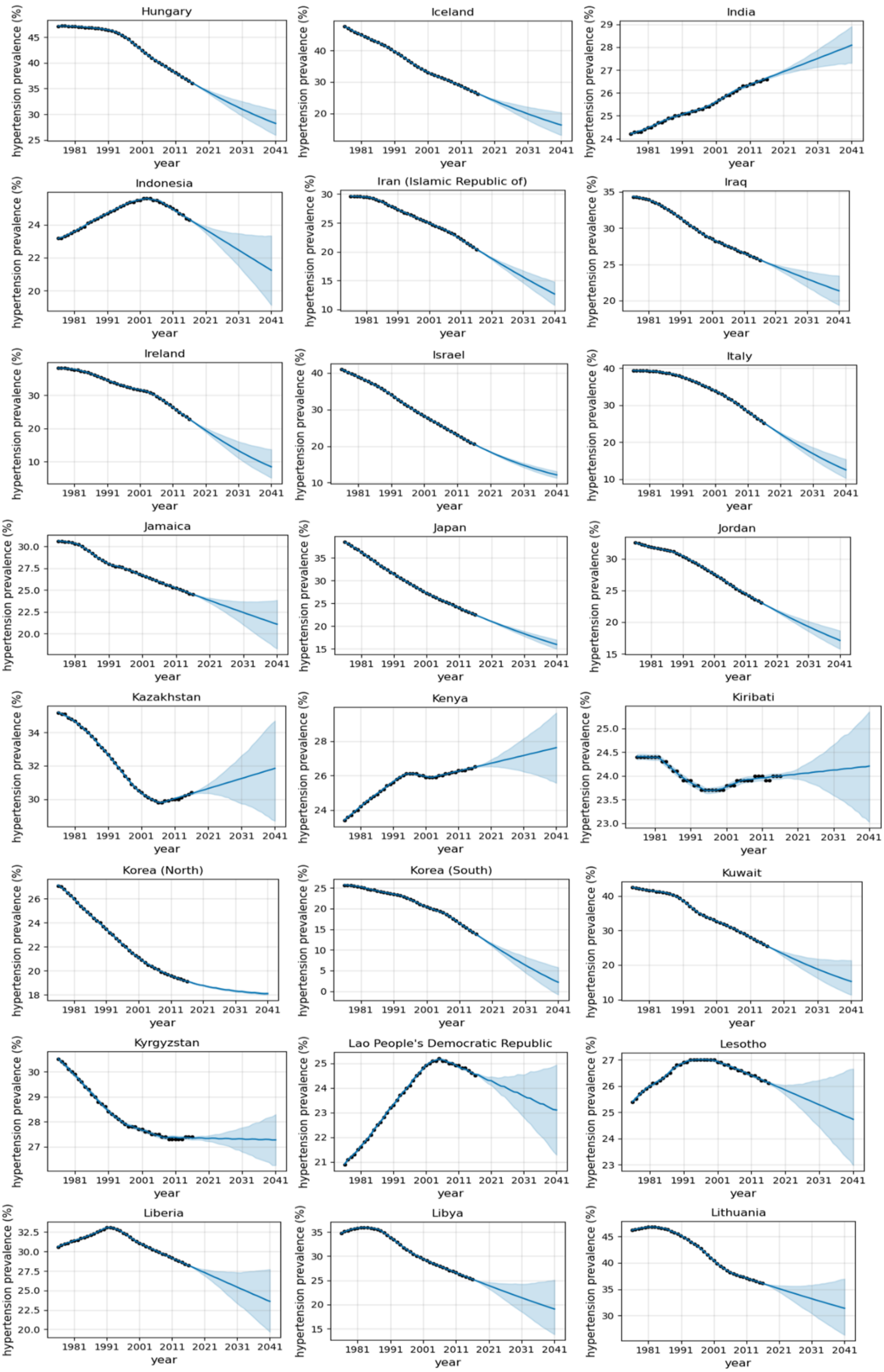

Fig. A1d. Plot of actual data (dotted) and fitted curve (95% CI) for hypertension prevalence (Male)

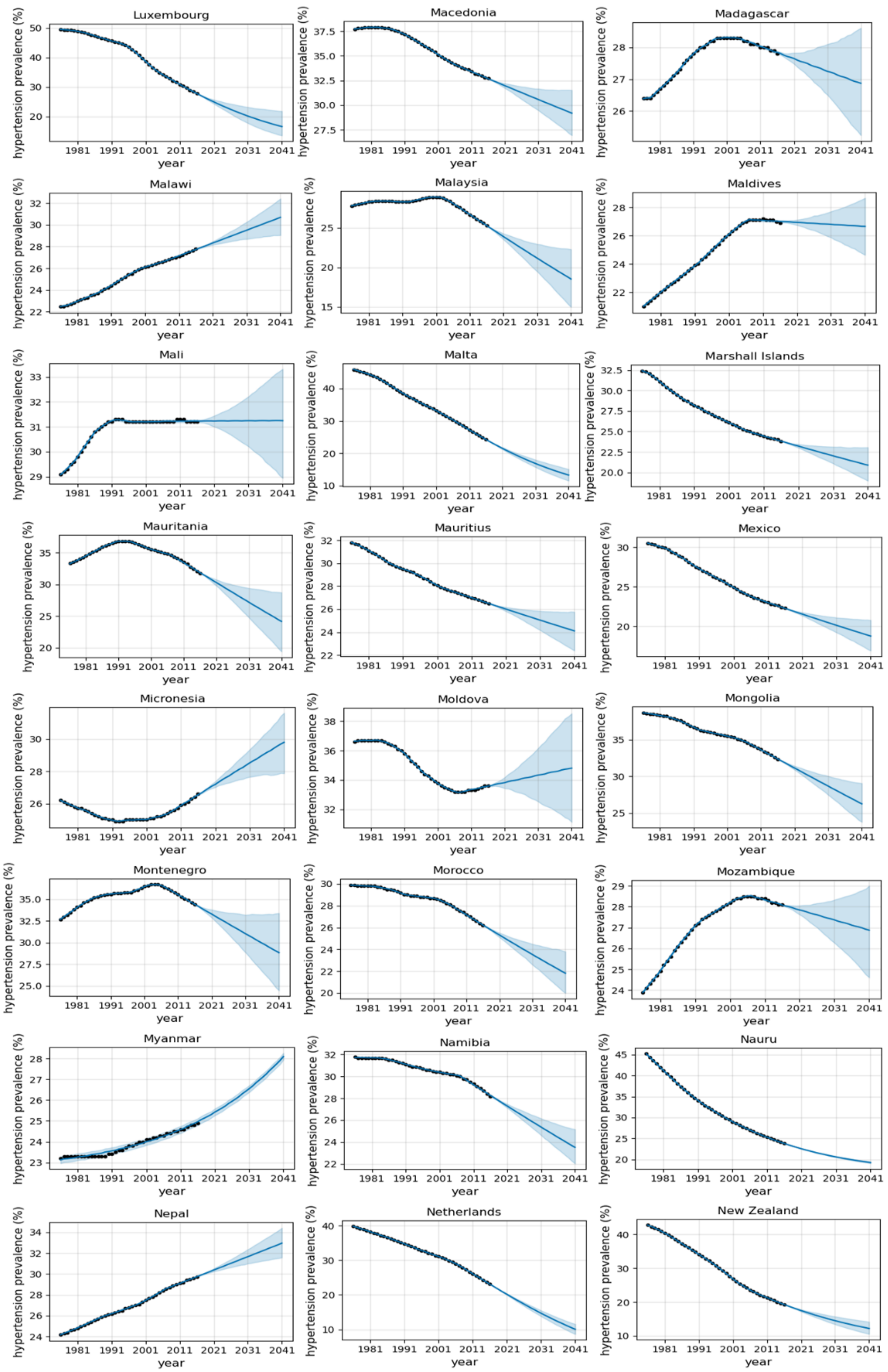

Fig. A1e. Plot of actual data (dotted) and fitted curve (95% CI) for hypertension prevalence (Male)

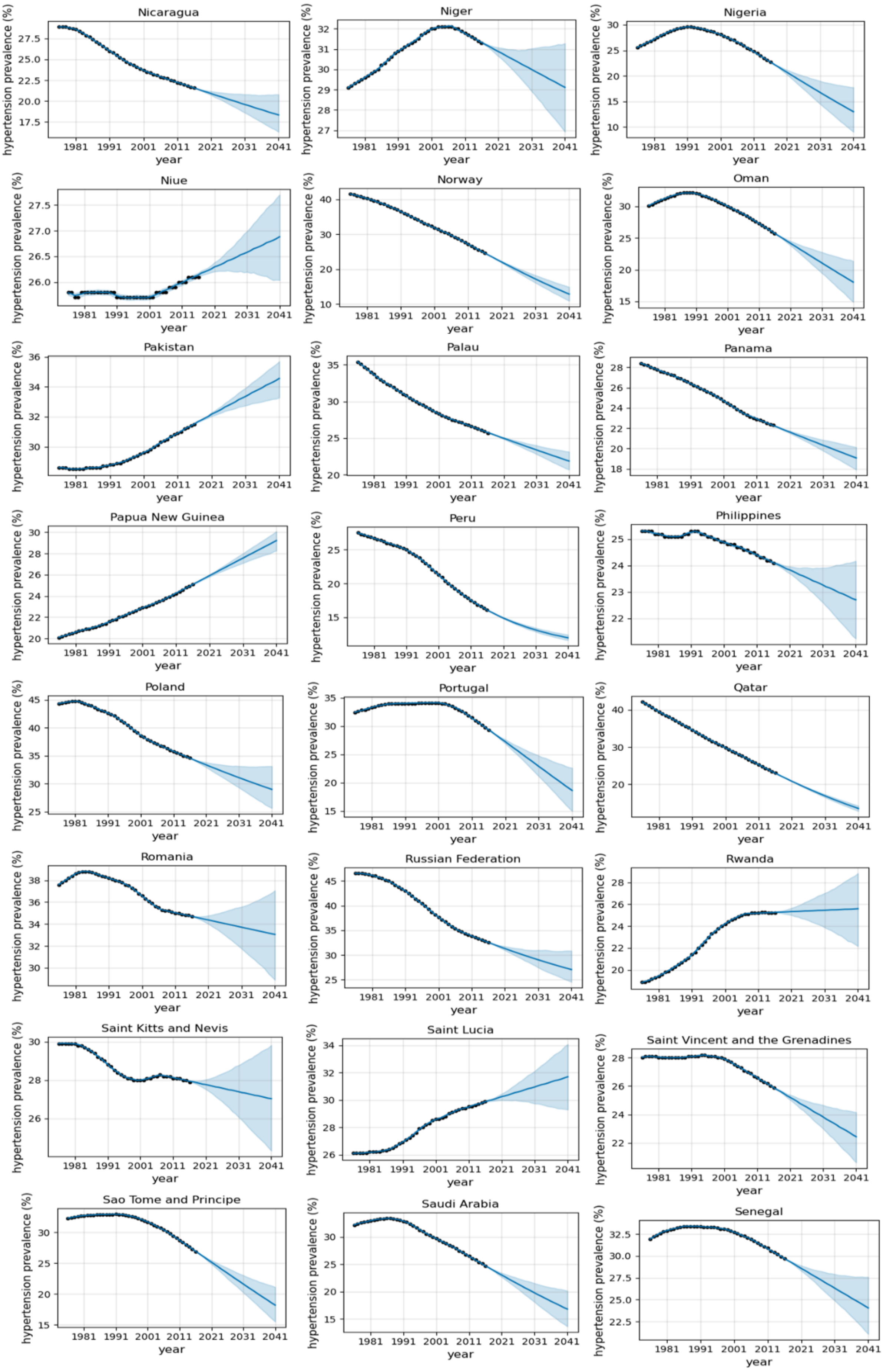

Fig. A1f. Plot of actual data (dotted) and fitted curve (95% CI) for hypertension prevalence (Male)

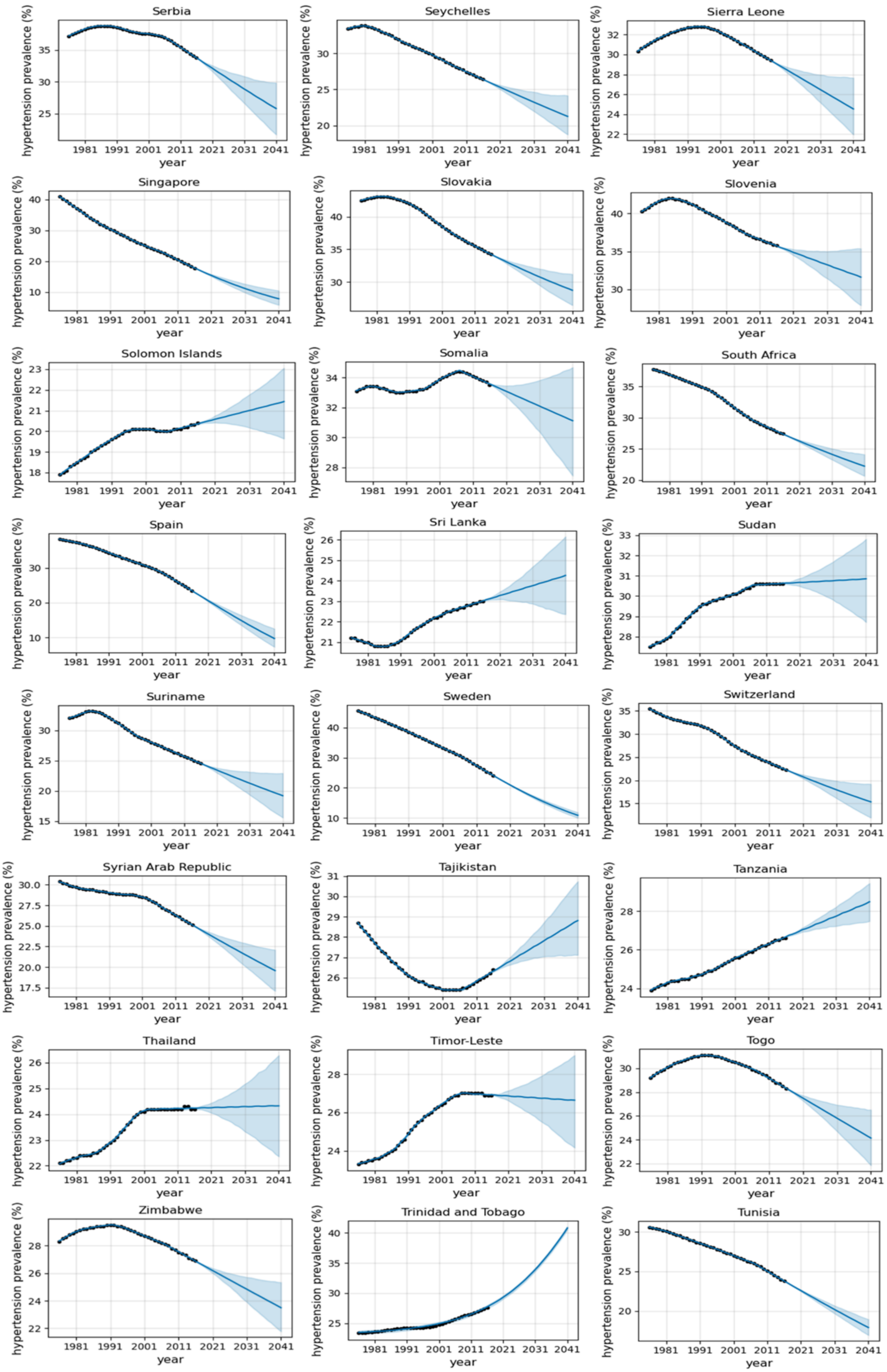

Fig. A1g. Plot of actual data (dotted) and fitted curve (95% CI) for hypertension prevalence (Male)

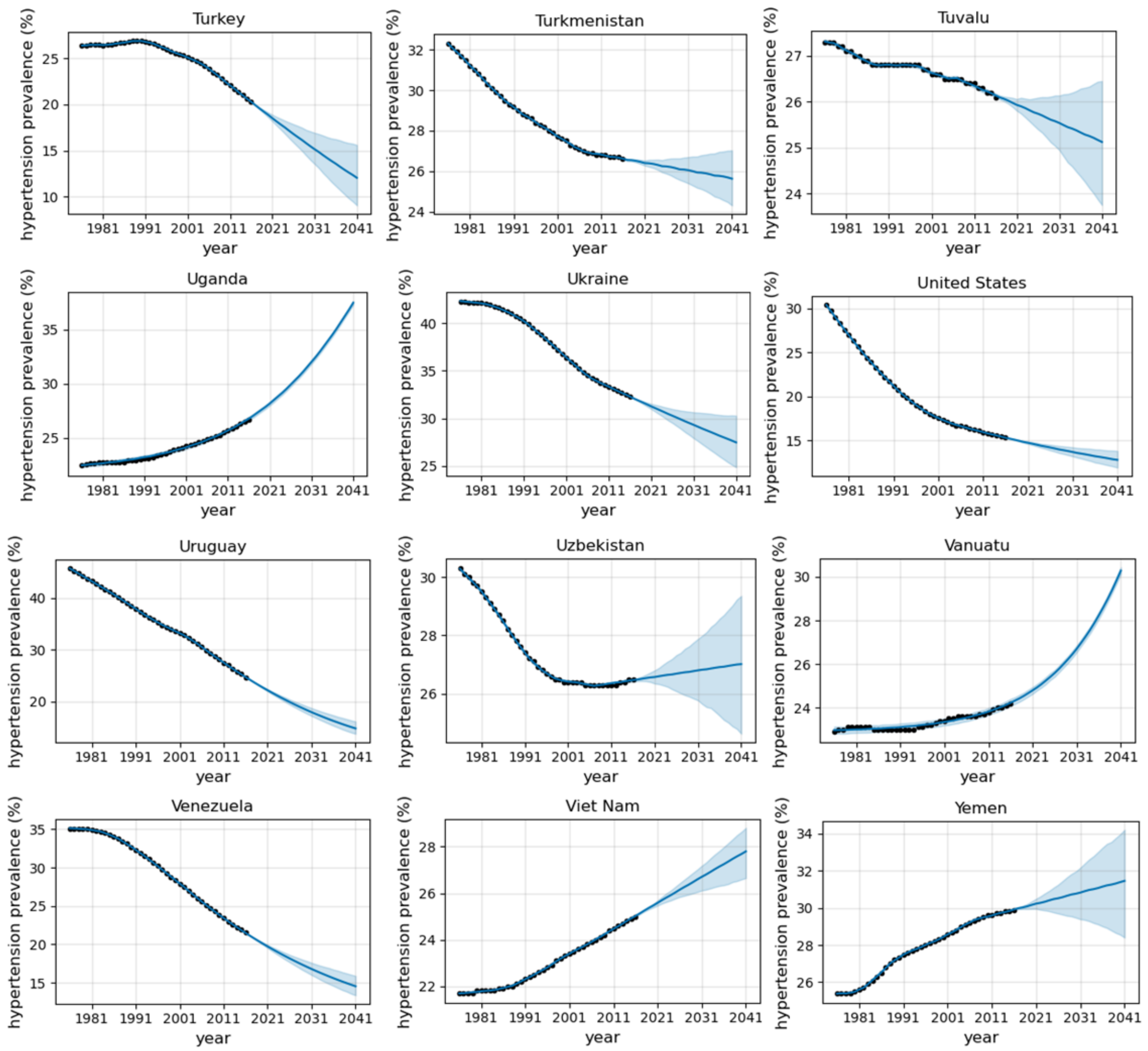

Fig. A1h. Plot of actual data (dotted) and fitted curve (95% CI) for hypertension prevalence (Male)

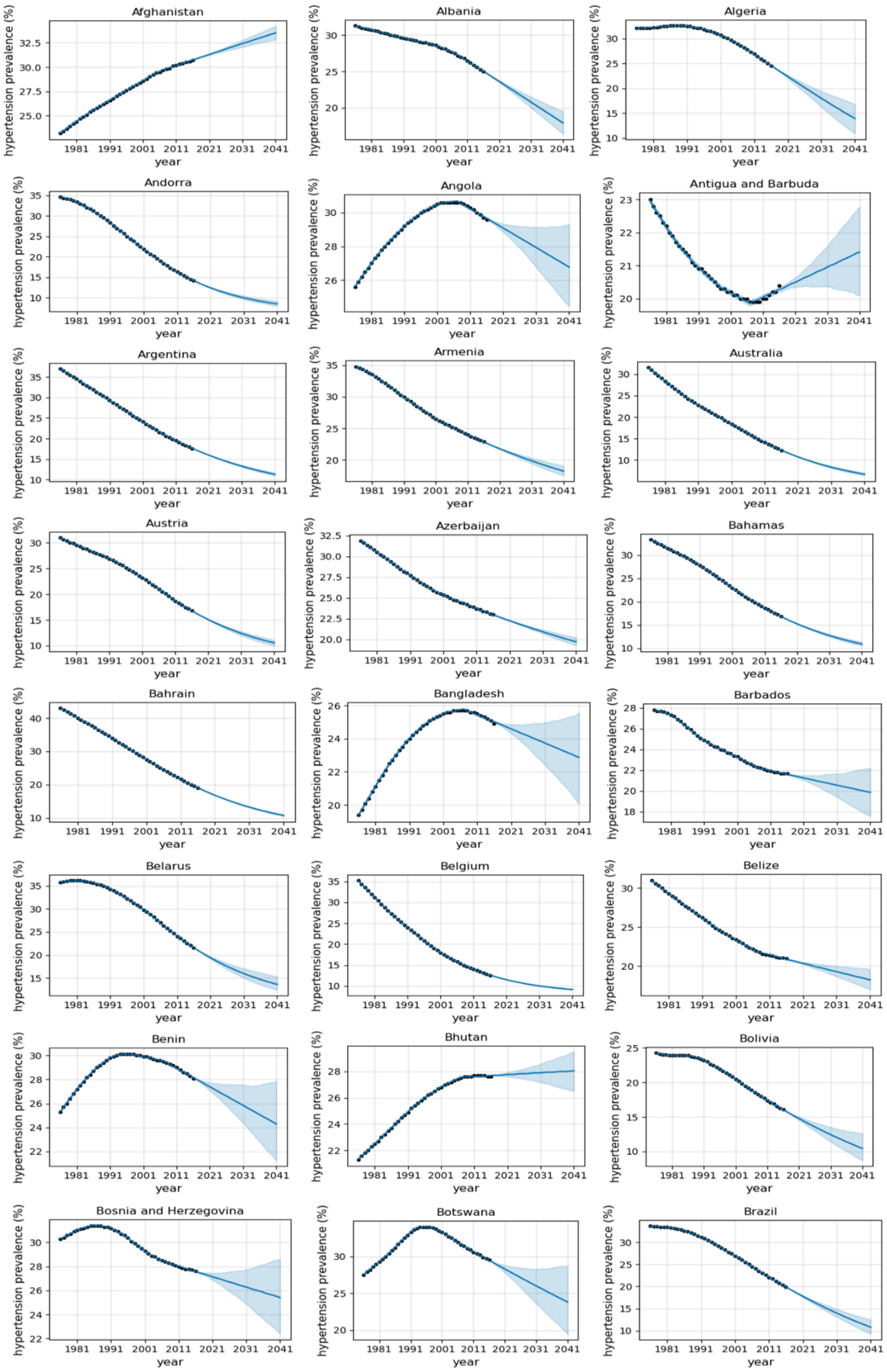

Fig. A2a. Plot of actual data (dotted) and fitted curve (95% CI) for hypertension prevalence (Female)

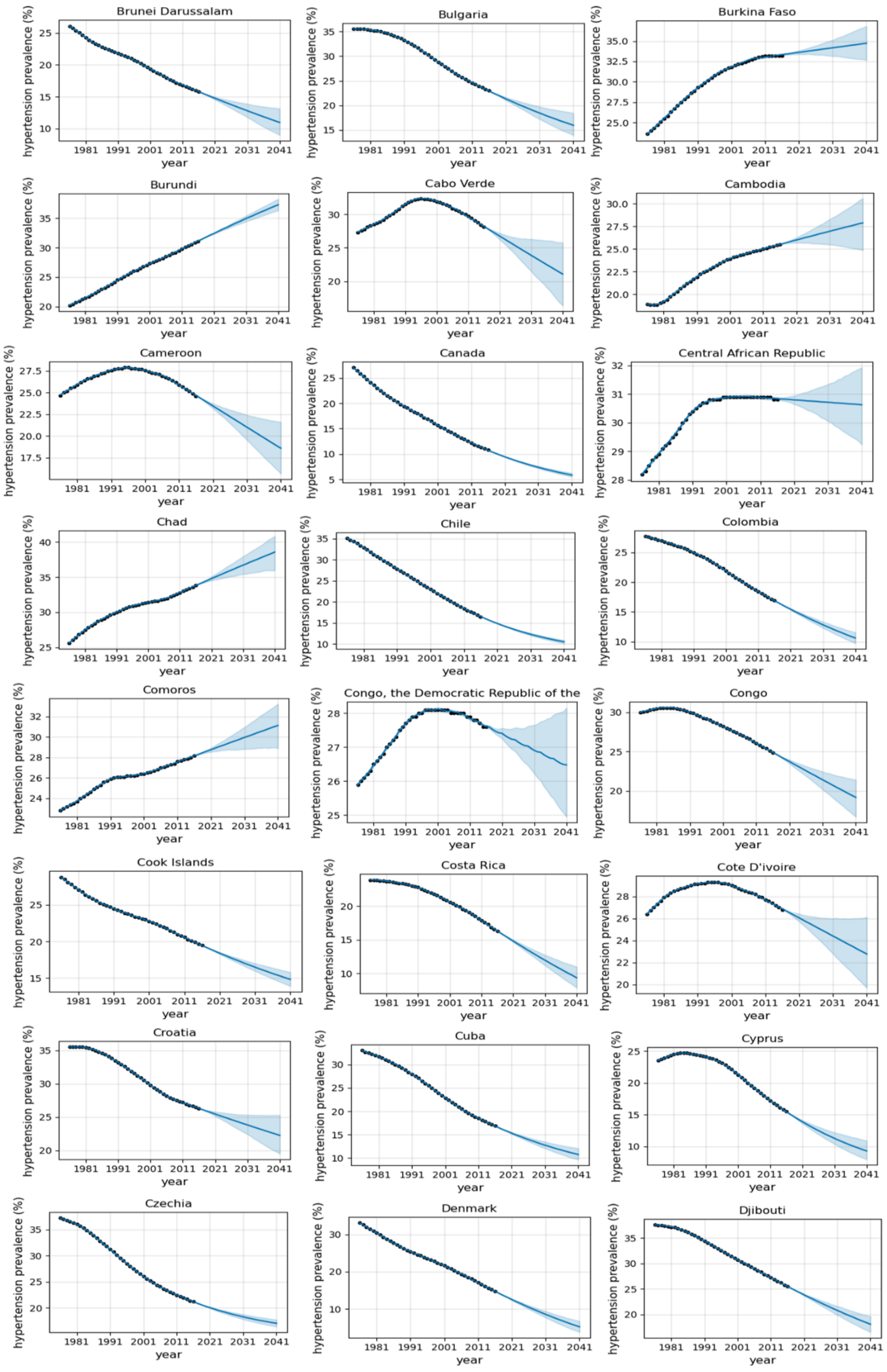

Fig. A2b. Plot of actual data (dotted) and fitted curve (95% CI) for hypertension prevalence (Female)

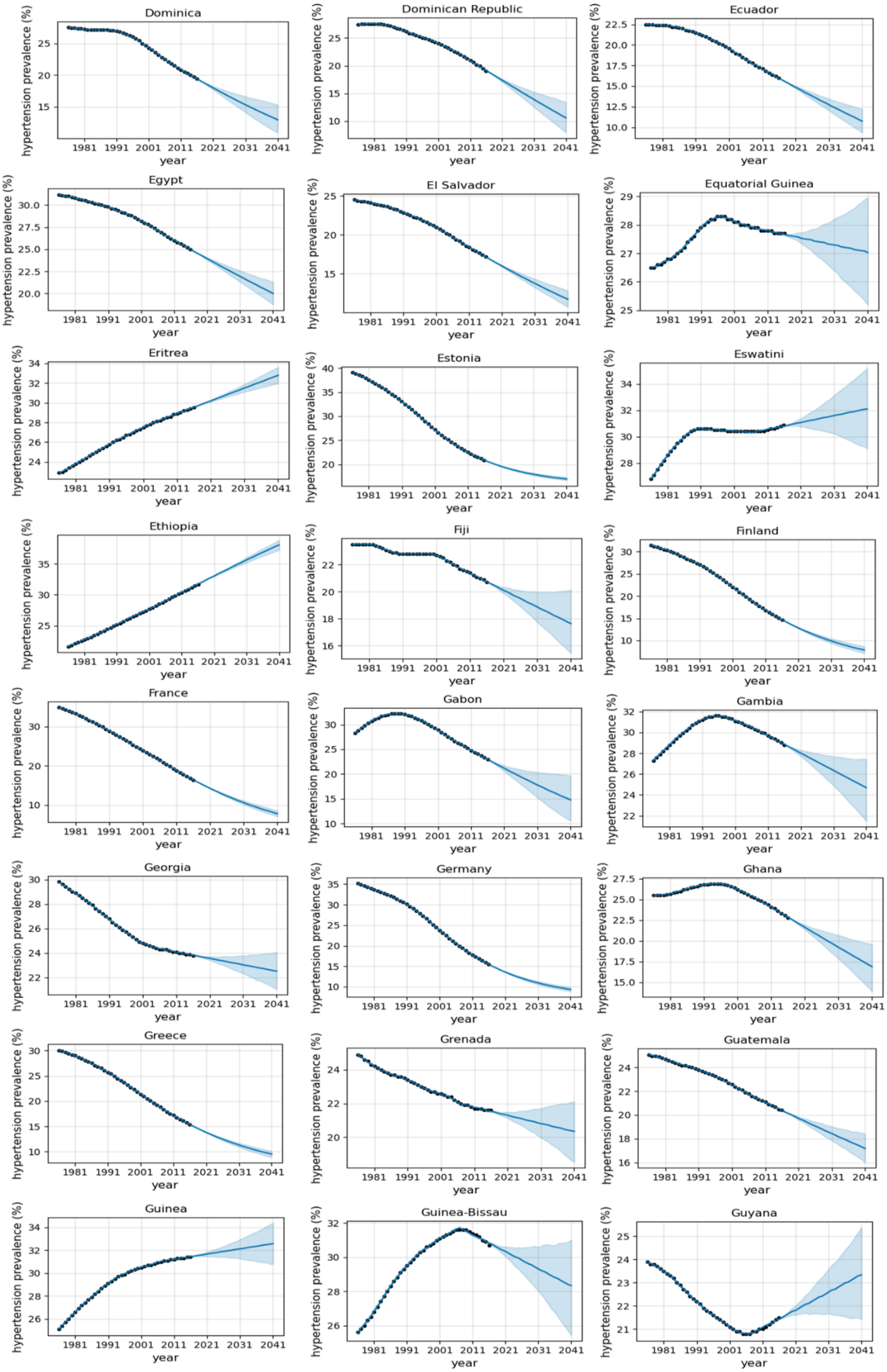

Fig. A2c. Plot of actual data (dotted) and fitted curve (95% CI) for hypertension prevalence (Female)

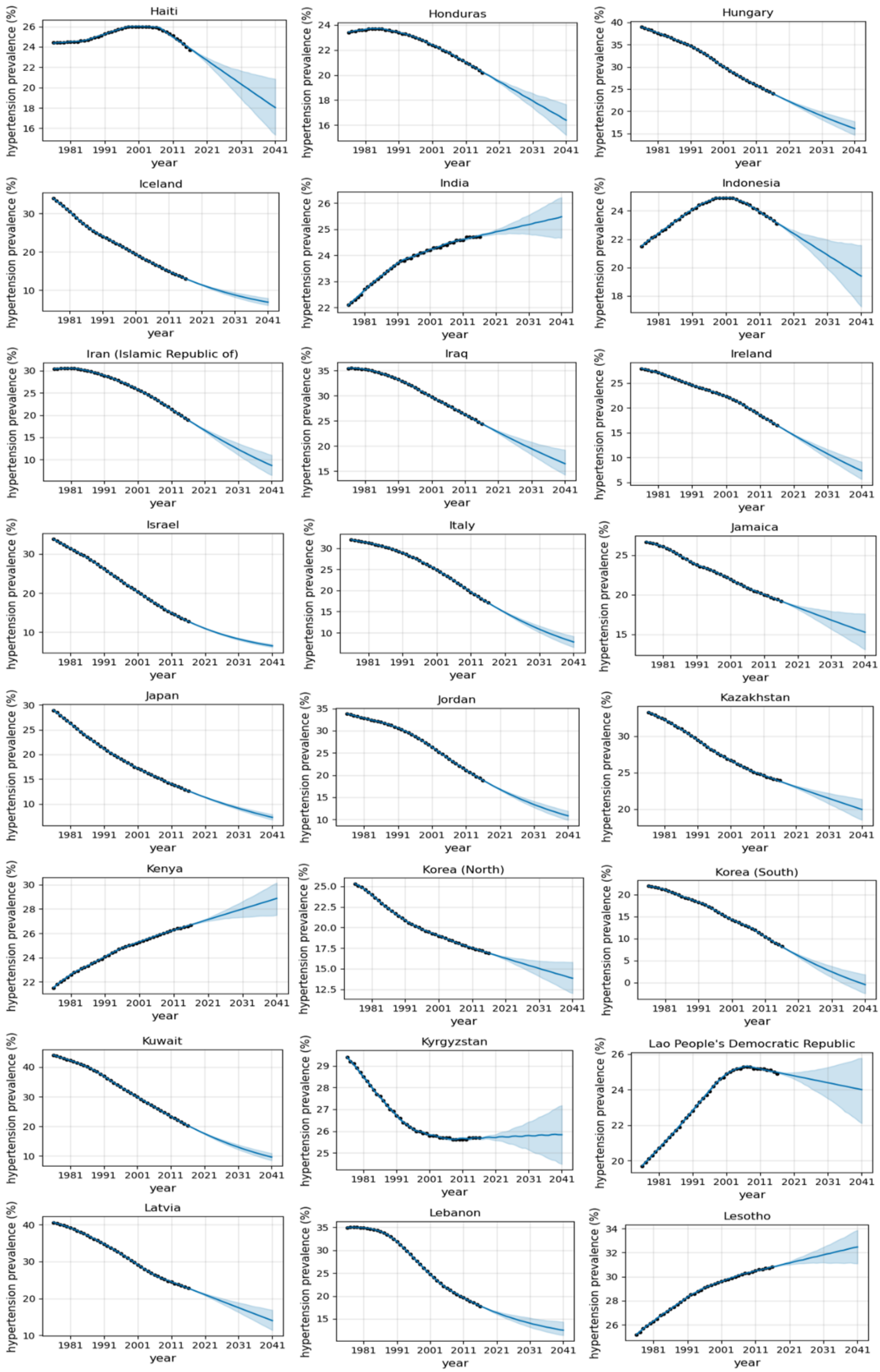

Fig. A2d. Plot of actual data (dotted) and fitted curve (95% CI) for hypertension prevalence (Female)

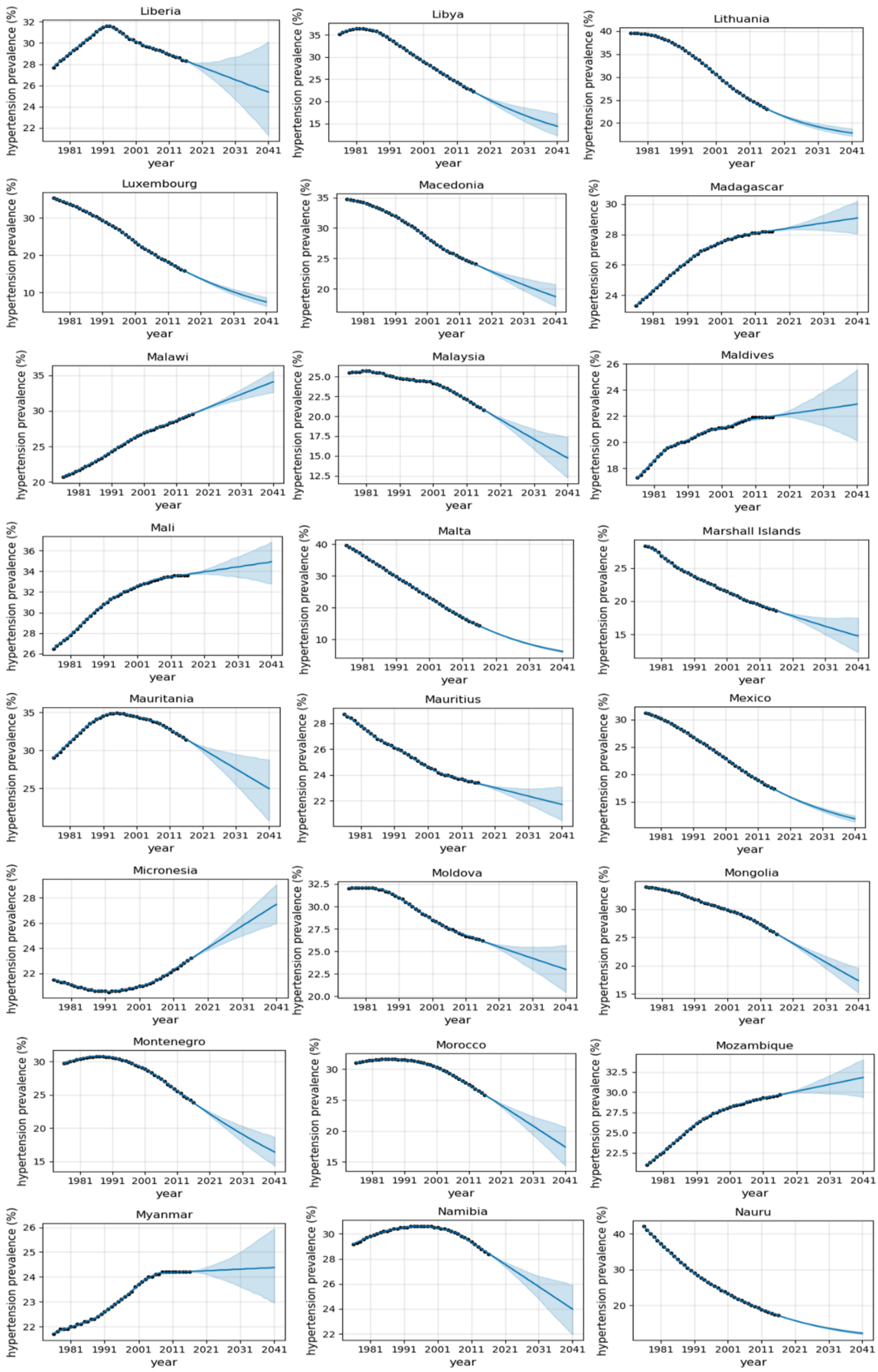

Fig. A2e. Plot of actual data (dotted) and fitted curve (95% CI) for hypertension prevalence (Female)

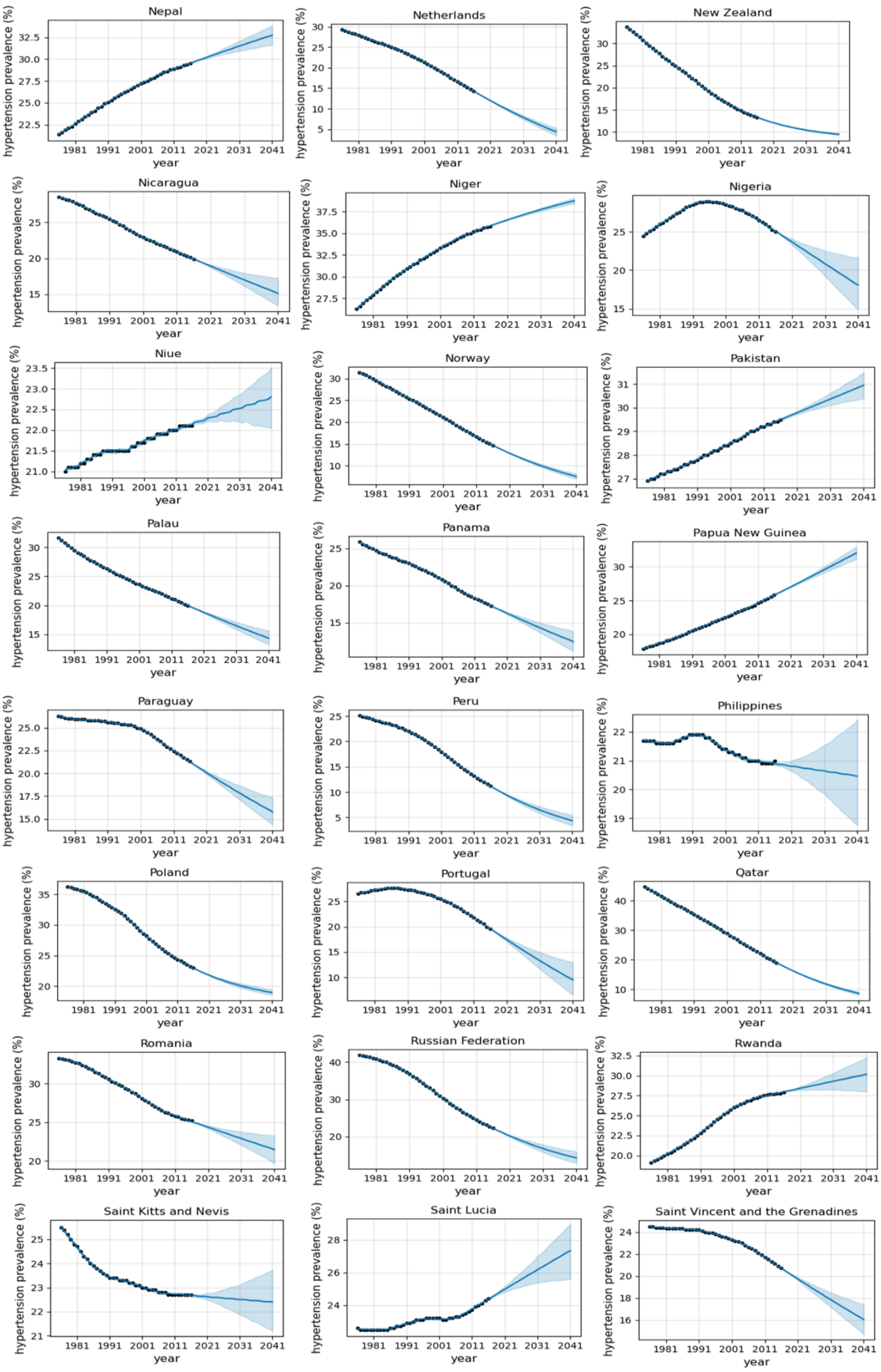

Fig. A2f. Plot of actual data (dotted) and fitted curve (95% CI) for hypertension prevalence (Female)

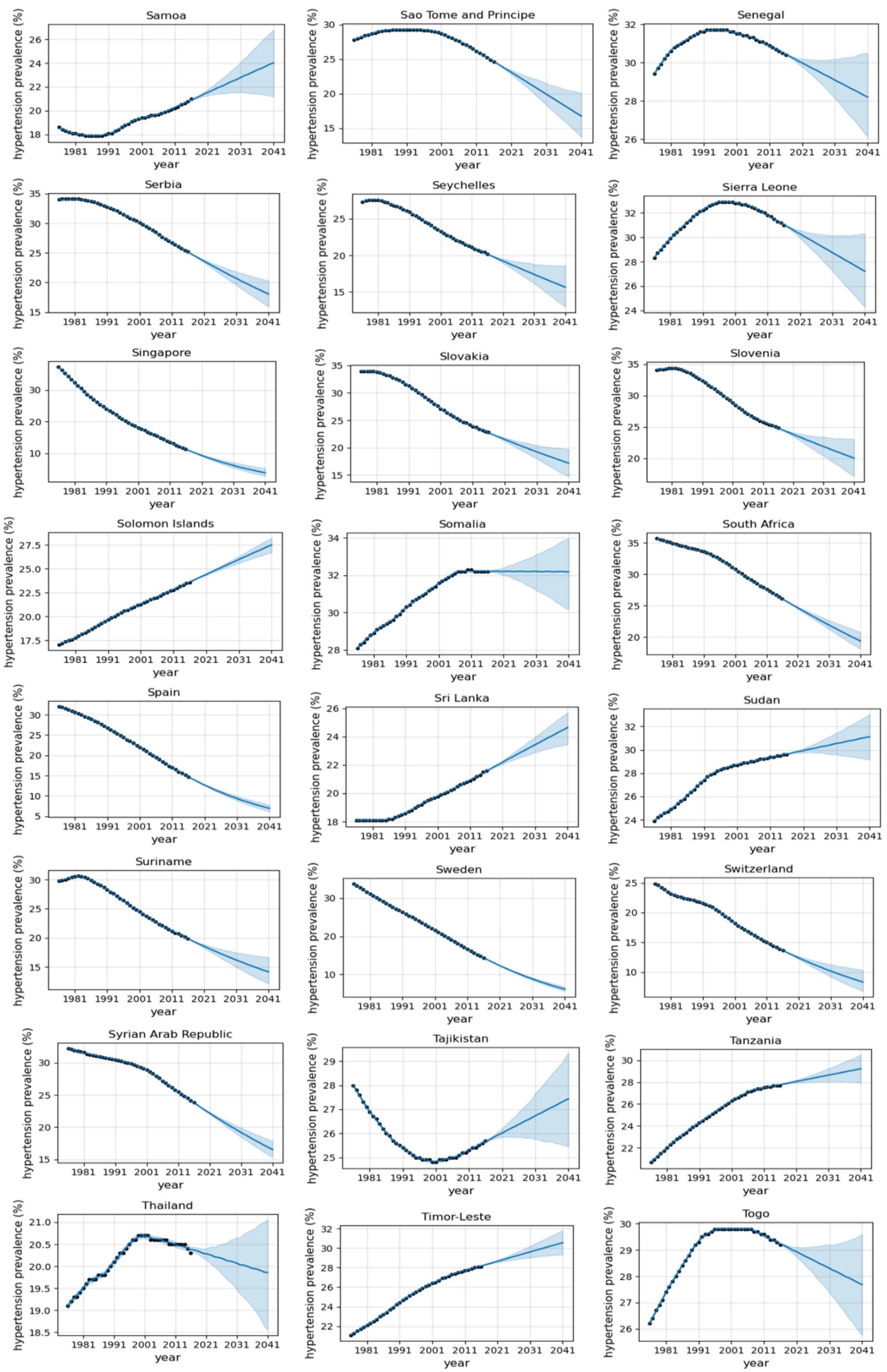

Fig. A2g. Plot of actual data (dotted) and fitted curve (95% CI) for hypertension prevalence (Female)

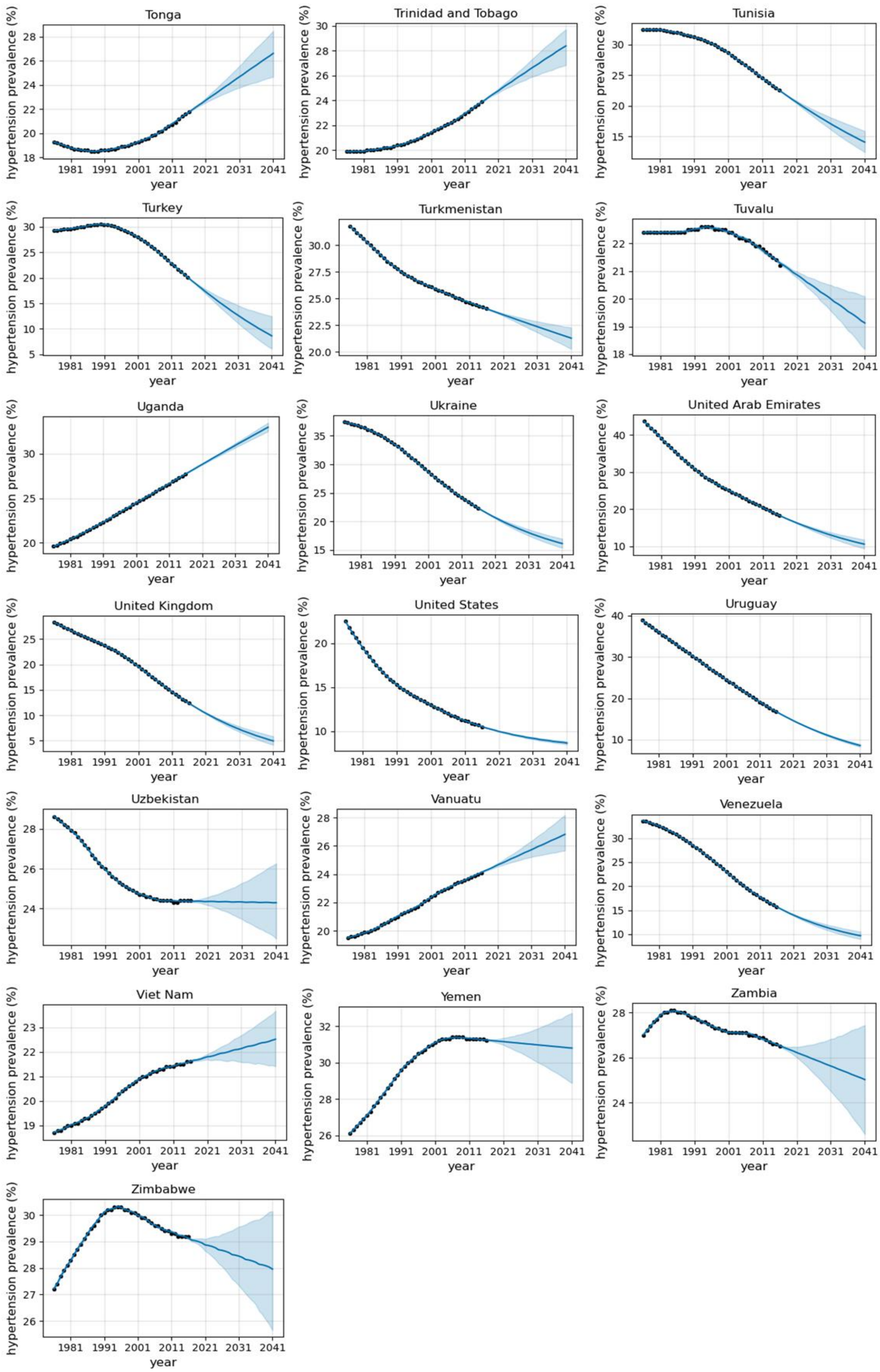

Fig. A2h. Plot of actual data (dotted) and fitted curve (95% CI) for hypertension prevalence (Female)

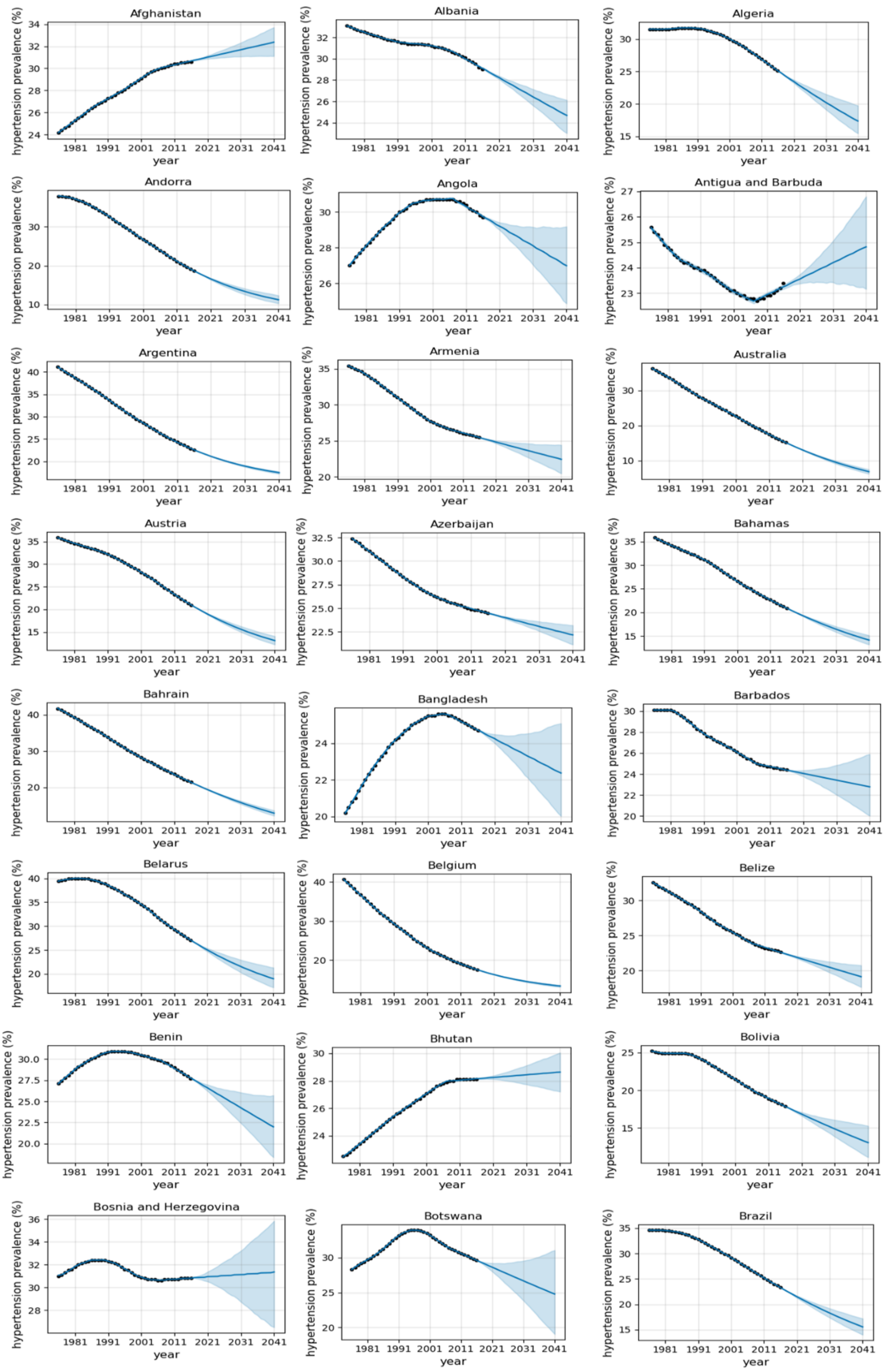

Fig. A3a. Plot of actual data (dotted) and fitted curve (95% CI) for hypertension prevalence (Both)

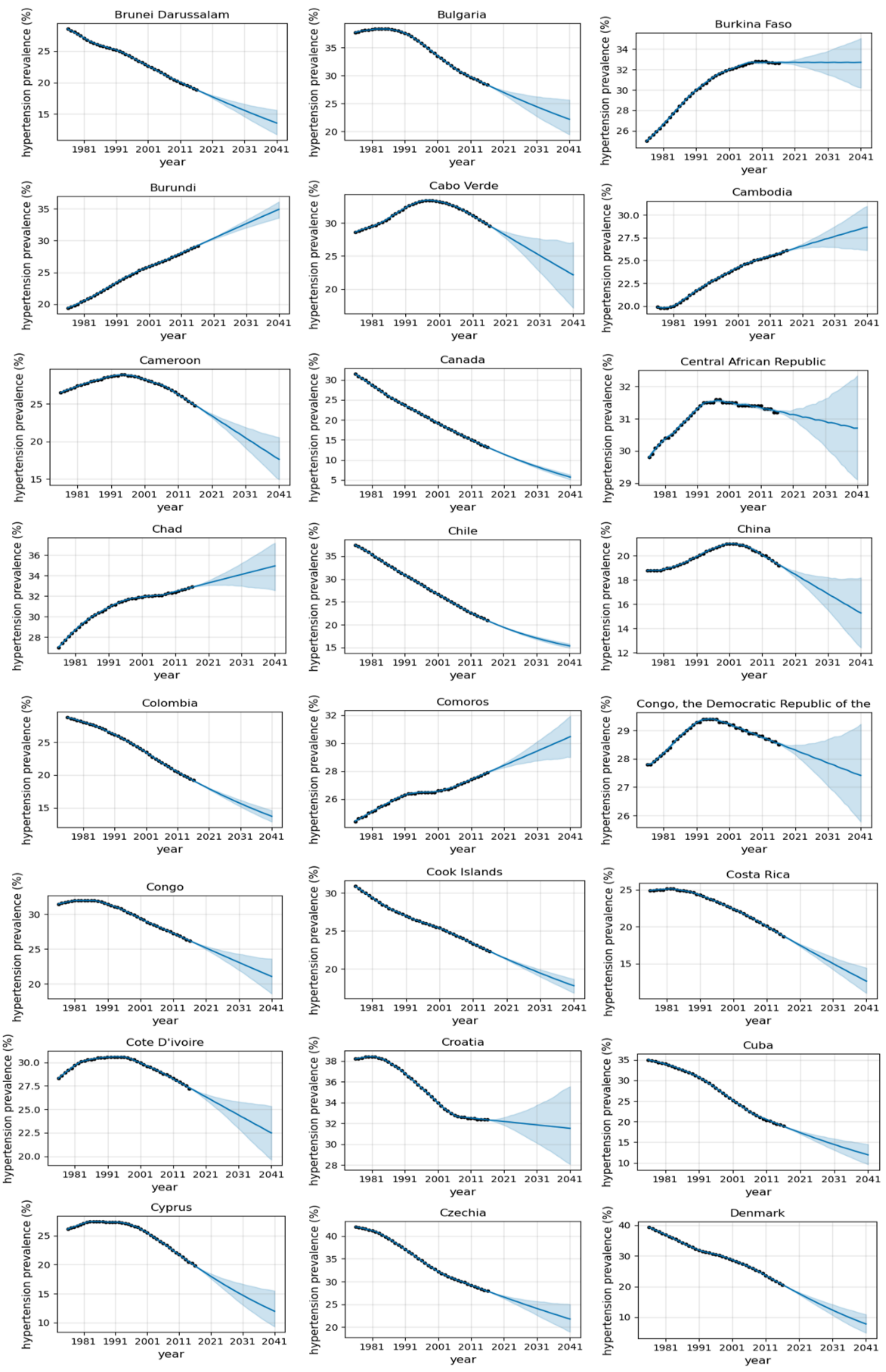

Fig. A3b. Plot of actual data (dotted) and fitted curve (95% CI) for hypertension prevalence (Both)

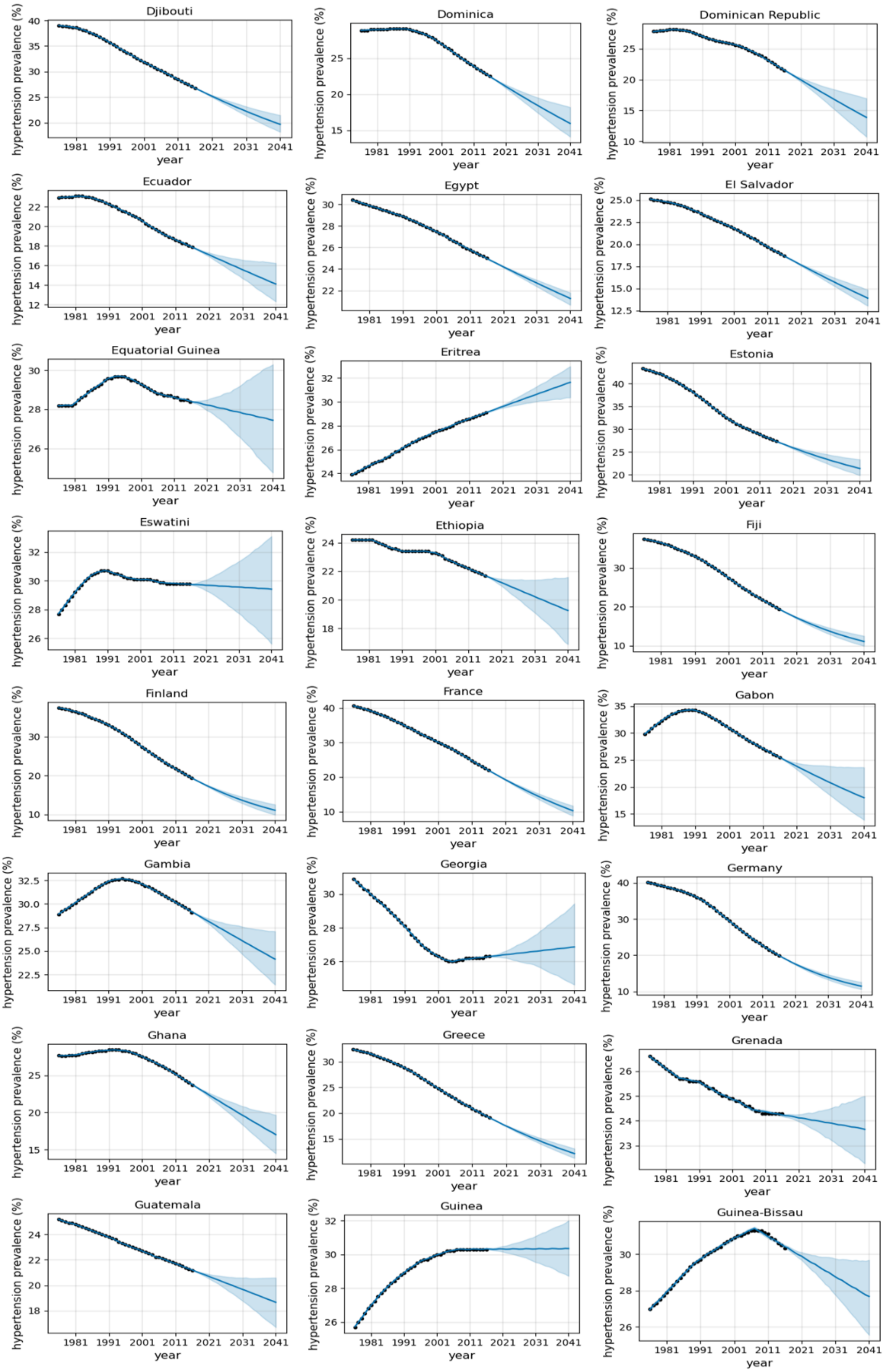

Fig. A3c. Plot of actual data (dotted) and fitted curve (95% CI) for hypertension prevalence (Both)

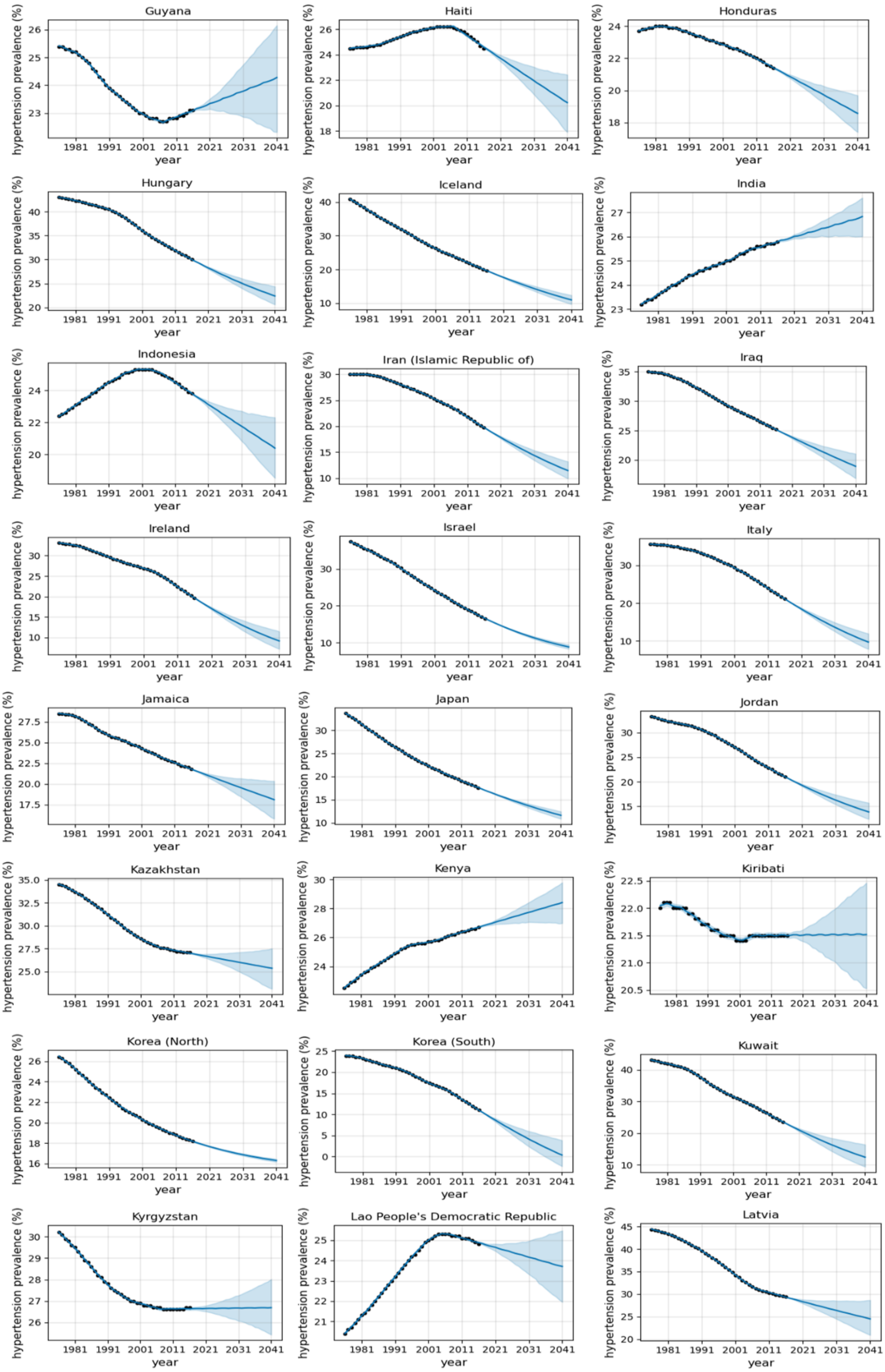

Fig. A3d. Plot of actual data (dotted) and fitted curve (95% CI) for hypertension prevalence (Both)

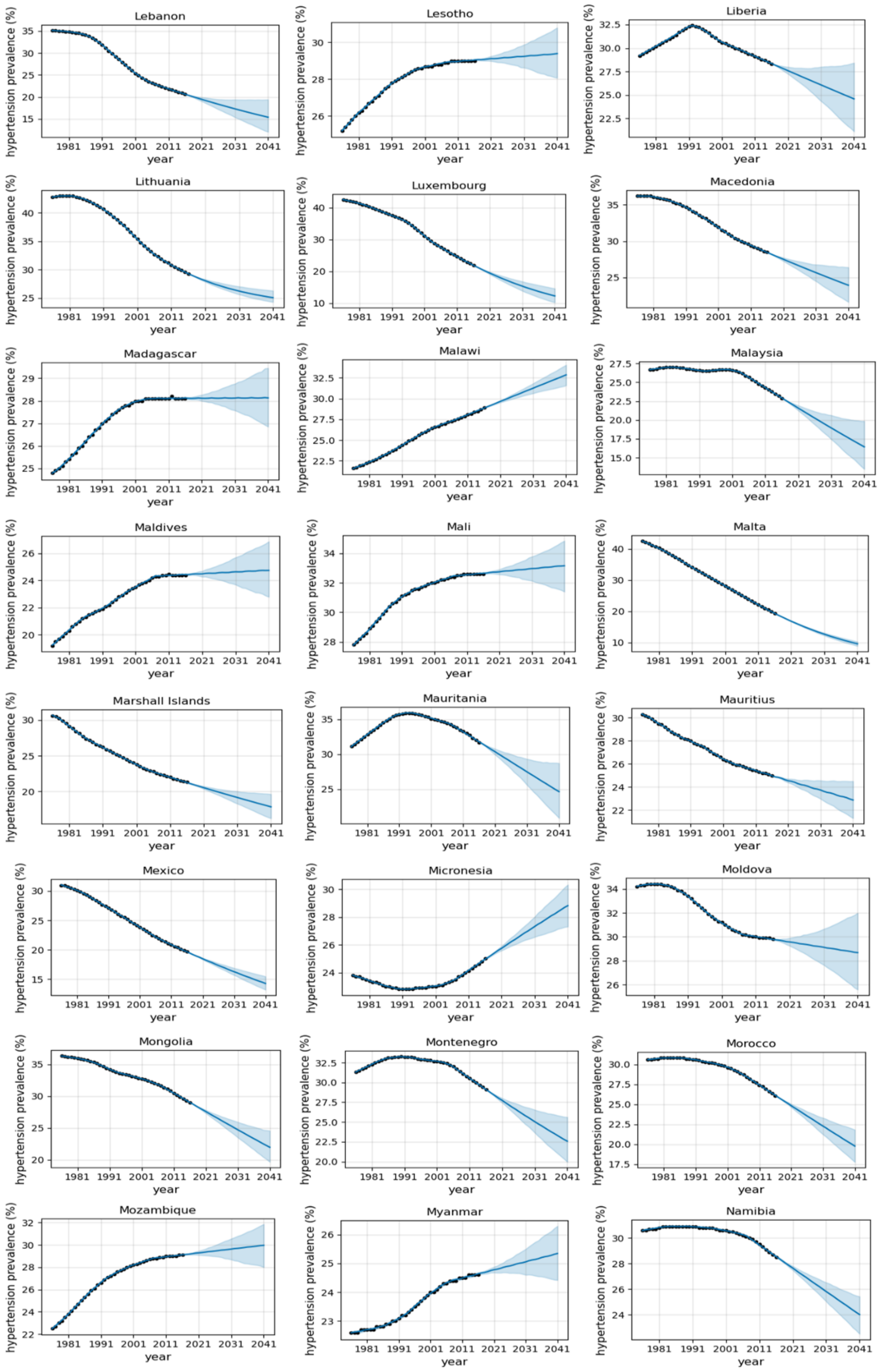

Fig. A3e. Plot of actual data (dotted) and fitted curve (95% CI) for hypertension prevalence (Both)

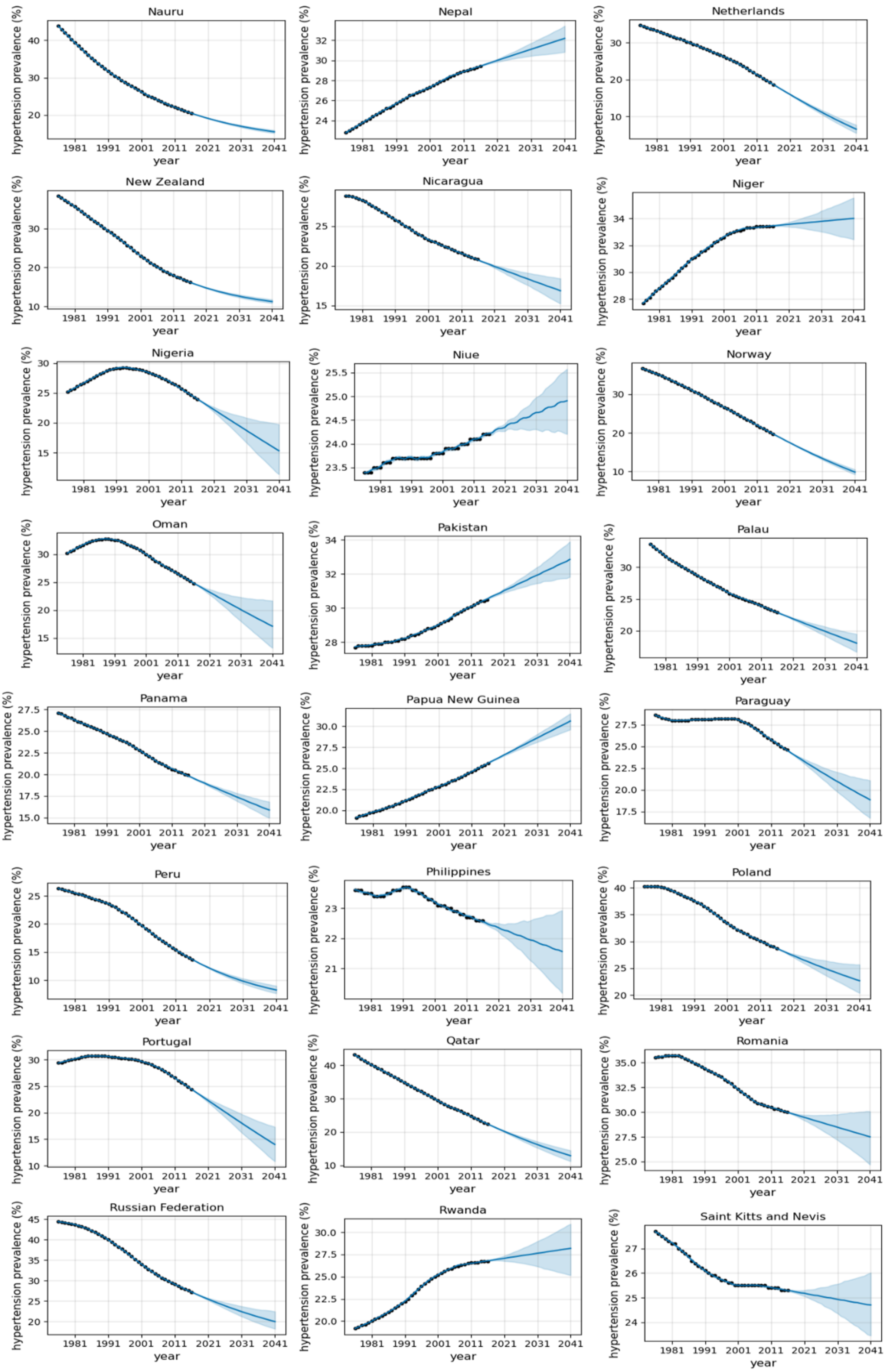

Fig. A3f. Plot of actual data (dotted) and fitted curve (95% CI) for hypertension prevalence (Both)

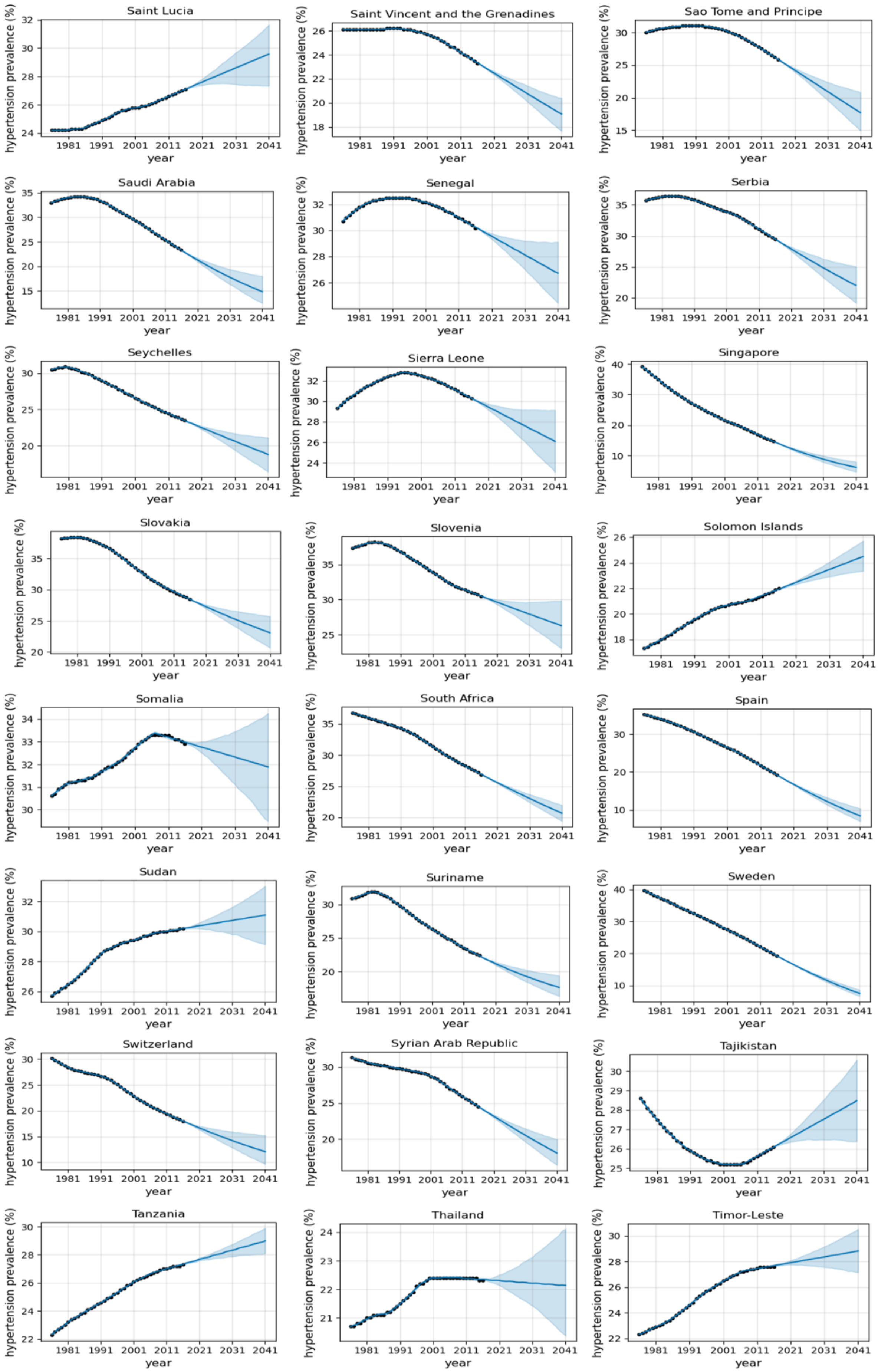

Fig. A3g. Plot of actual data (dotted) and fitted curve (95% CI) for hypertension prevalence (Both)

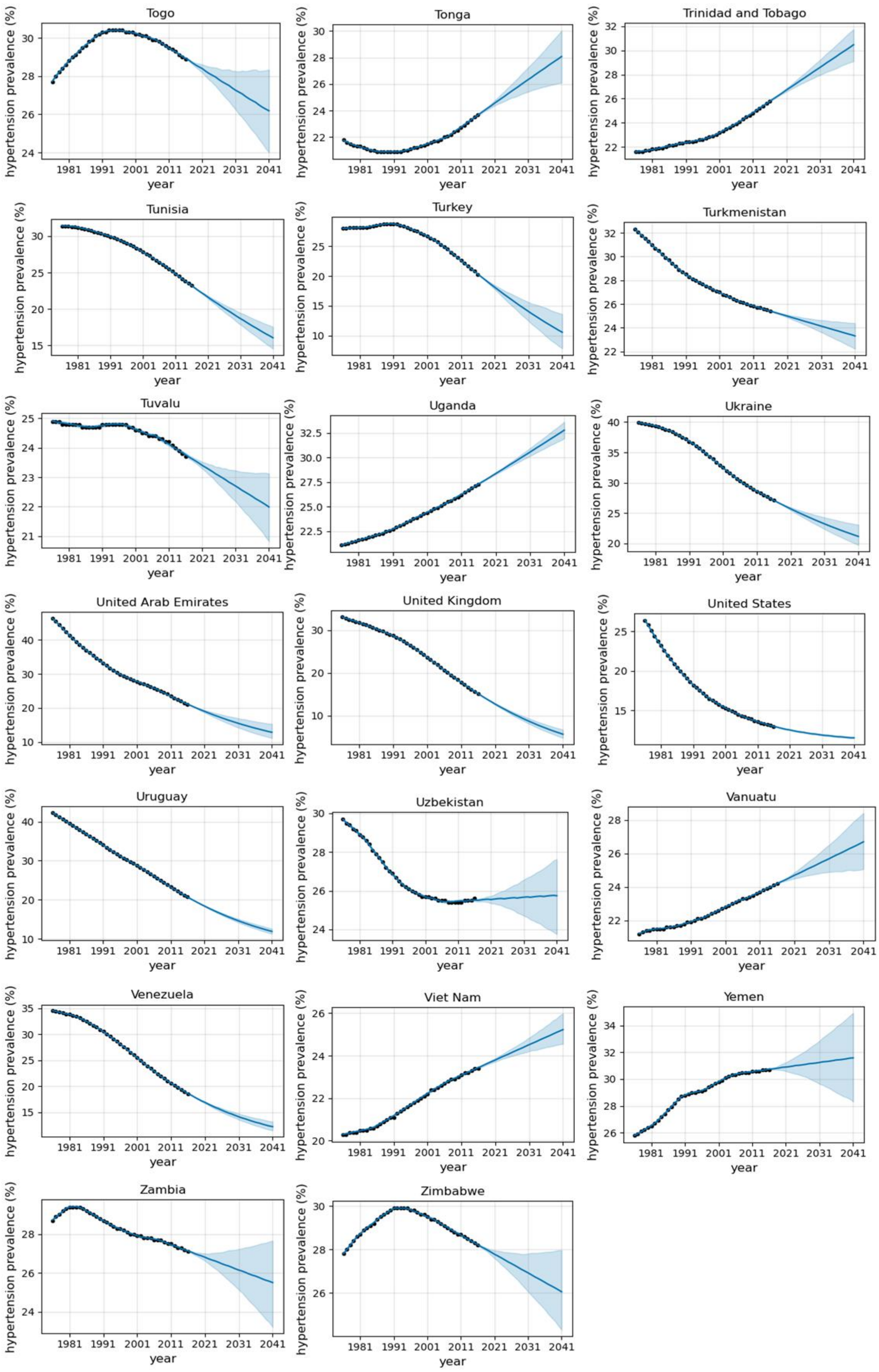

Fig. A3h. Plot of actual data (dotted) and fitted curve (95% CI) for hypertension prevalence (Both)

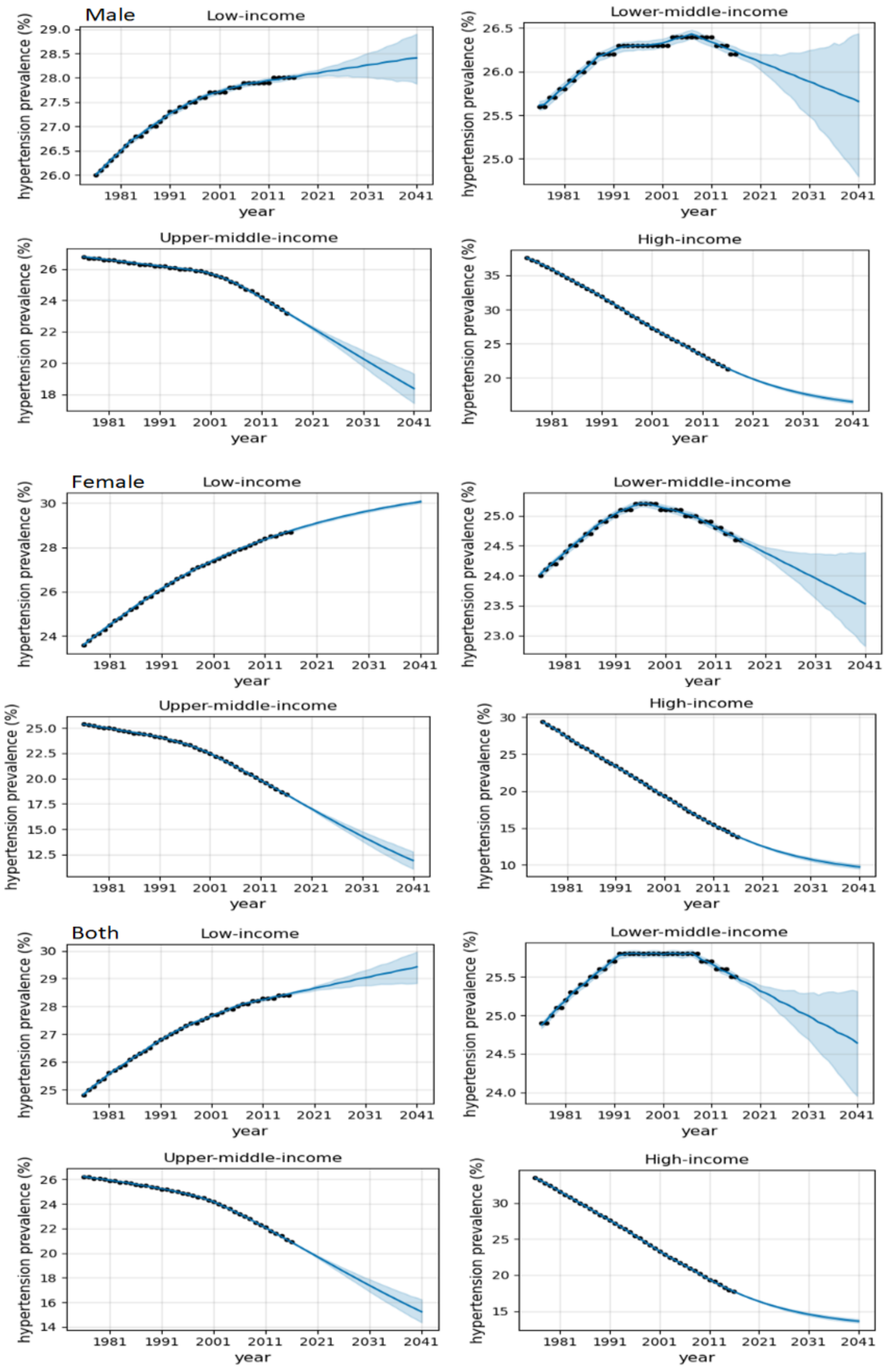

Fig. A4a. Plot of actual data and fitted curve (95% CI) for hypertension prevalence (Income groups)

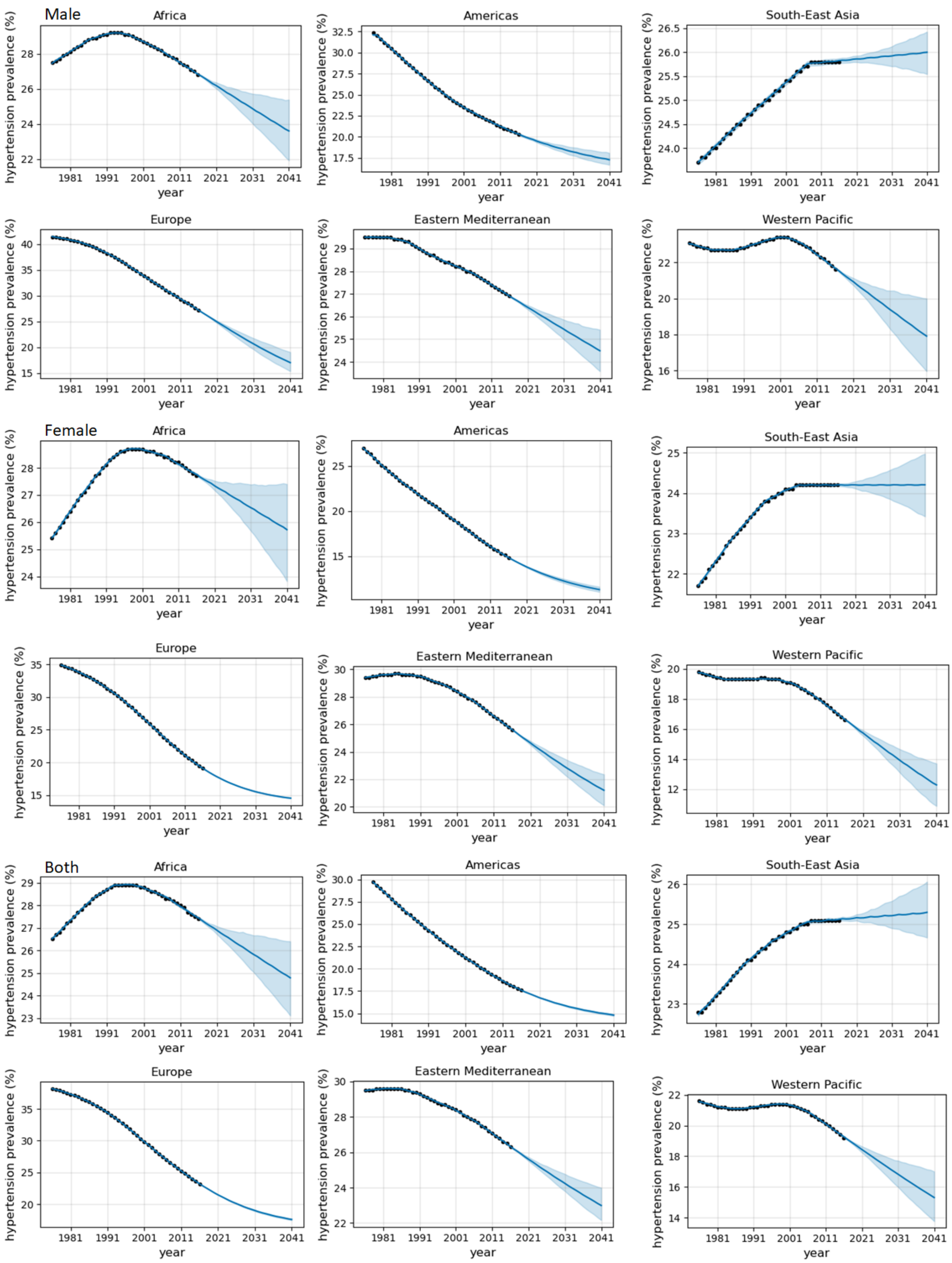

Fig. A4b. Plot of actual data and fitted curve (95% CI) for hypertension prevalence (Regions)

Table A4. Changes in hypertension prevalence cluster patterns

No.	Country/territory	2010	2015	2040
1	Afghanistan	1	1	1
2	Angola	1	1	1
3	Bhutan	2	1	1
4	Bosnia and Herzegovina	1	1	1
5	Burkina Faso	1	1	1
6	Burundi	2	1	1
7	Cambodia	2	2	1
8	Central African Republic	1	1	1
9	Chad	1	1	1
10	Comoros	2	1	1
11	Congo, the Democratic Republic of the	2	1	1
12	Croatia	1	1	1
13	Equatorial Guinea	2	1	1
14	Eritrea	2	1	1
15	Eswatini	2	1	1
16	Ethiopia	2	1	1
17	Guinea	1	1	1
18	Guinea-Bissau	1	1	1
19	India	2	2	1
20	Kenya	2	2	1
21	Kyrgyzstan	2	2	1
22	Lesotho	2	1	1
23	Madagascar	2	1	1
24	Malawi	2	1	1
25	Mali	1	1	1
26	Micronesia	2	2	1
27	Moldova	1	1	1
28	Mozambique	2	1	1
29	Myanmar	2	2	1
30	Nepal	2	1	1
31	Niger	1	1	1
32	Pakistan	1	1	1
33	Papua New Guinea	2	2	1
34	Romania	1	1	1
35	Rwanda	2	2	1
36	Saint Lucia	2	1	1
37	Senegal	1	1	1
38	Sierra Leone	1	1	1
39	South Africa	2	2	1
40	Sudan	1	1	1
41	Tajikistan	2	2	1
42	Tanzania	2	2	1
43	Timor-Leste	2	1	1
44	Togo	2	1	1
45	Trinidad and Tobago	2	2	1
46	Uganda	2	1	1
47	Vanuatu	2	2	1

No.	Country/territory	2010	2015	2040
48	Yemen	1	1	1
49	Zimbabwe	2	1	1
50	Albania	1	1	2
51	Antigua and Barbuda	3	2	2
52	Argentina	3	2	2
53	Armenia	2	2	2
54	Azerbaijan	2	2	2
55	Bangladesh	2	2	2
56	Barbados	2	2	2
57	Belarus	1	1	2
58	Belize	3	2	2
59	Benin	2	1	2
60	Botswana	1	1	2
61	Bulgaria	1	1	2
62	Cabo Verde	1	1	2
63	Congo	2	2	2
64	Cook Islands	3	2	2
65	Cote D'ivoire	2	1	2
66	Czechia	1	1	2
67	Djibouti	2	1	2
68	Egypt	2	2	2
69	Estonia	1	1	2
70	Fiji	3	2	2
71	Gambia	1	1	2
72	Grenada	3	2	2
73	Guatemala	3	3	2
74	Haiti	2	2	2
75	Honduras	3	2	2
76	Hungary	1	1	2
77	Indonesia	2	2	2
78	Iraq	2	2	2
79	Jamaica	3	2	2
80	Kazakhstan	2	1	2
81	Lao People's Democratic Republic	2	2	2
82	Liberia	2	1	2
83	Lithuania	1	1	2
84	Macedonia	1	1	2
85	Maldives	2	2	2
86	Marshall Islands	3	3	2
87	Mauritania	1	1	2
88	Mauritius	2	2	2
89	Mongolia	1	1	2
90	Montenegro	1	1	2
91	Morocco	2	2	2
92	Namibia	2	1	2
93	Niue	3	2	2
94	Palau	3	2	2
95	Philippines	3	2	2

No.	Country/territory	2010	2015	2040
96	Poland	1	1	2
97	Russian Federation	1	1	2
98	Saint Kitts and Nevis	2	2	2
99	Saint Vincent and the Grenadines	3	2	2
100	Serbia	1	1	2
101	Seychelles	3	2	2
102	Slovakia	1	1	2
103	Slovenia	1	1	2
104	Solomon Islands	3	2	2
105	Somalia	1	1	2
106	Sri Lanka	3	2	2
107	Syrian Arab Republic	2	2	2
108	Thailand	3	2	2
109	Turkmenistan	2	2	2
110	Tuvalu	3	2	2
111	Ukraine	1	1	2
112	Uzbekistan	2	2	2
113	Viet Nam	3	2	2
114	Algeria	2	2	3
115	Andorra	3	3	3
116	Australia	3	3	3
117	Austria	3	3	3
118	Bahamas	3	3	3
119	Bahrain	3	3	3
120	Belgium	3	3	3
121	Bolivia	3	3	3
122	Brazil	2	2	3
123	Brunei Darussalam	3	3	3
124	Cameroon	2	2	3
125	Canada	3	3	3
126	Chile	3	3	3
127	Colombia	3	3	3
128	Costa Rica	3	3	3
129	Cuba	3	3	3
130	Cyprus	3	3	3
131	Denmark	3	3	3
132	Dominica	3	2	3
133	Dominican Republic	3	3	3
134	Ecuador	3	3	3
135	El Salvador	3	3	3
136	Finland	3	3	3
137	France	3	2	3
138	Germany	3	3	3
139	Ghana	2	2	3
140	Greece	3	3	3
141	Iceland	3	3	3
142	Iran (Islamic Republic of)	3	3	3
143	Ireland	3	3	3

No.	Country/territory	2010	2015	2040
144	Israel	3	3	3
145	Italy	3	3	3
146	Japan	3	3	3
147	Jordan	3	3	3
148	Korea (North)	3	3	3
149	Kuwait	2	2	3
150	Libya	2	2	3
151	Luxembourg	3	3	3
152	Malaysia	2	2	3
153	Malta	3	3	3
154	Mexico	3	3	3
155	Nauru	3	3	3
156	Netherlands	3	3	3
157	New Zealand	3	3	3
158	Nicaragua	3	3	3
159	Nigeria	2	2	3
160	Norway	3	3	3
161	Panama	3	3	3
162	Peru	3	3	3
163	Portugal	2	2	3
164	Qatar	3	3	3
165	Sao Tome and Principe	2	2	3
166	Singapore	3	3	3
167	Spain	3	3	3
168	Suriname	3	2	3
169	Sweden	3	3	3
170	Switzerland	3	3	3
171	Tunisia	2	2	3
172	Turkey	3	3	3
173	United Kingdom	3	3	3
174	United States	3	3	3
175	Uruguay	3	3	3
176	Venezuela	3	3	3
